# Low birthweight in patients with type 2 diabetes is associated with elevated risk of cardiovascular events and mortality

**DOI:** 10.1007/s00125-024-06170-z

**Published:** 2024-05-22

**Authors:** Aleksander L. Hansen, Charlotte Brøns, Leonie M. Engelhard, Mette K. Andersen, Torben Hansen, Jens S. Nielsen, Peter Vestergaard, Kurt Højlund, Niels Jessen, Michael H. Olsen, Henrik T. Sørensen, Reimar W. Thomsen, Allan Vaag

**Affiliations:** 1 Steno Diabetes Center Copenhagen, Herlev, Denmark; 2 Department of Clinical Epidemiology, Aarhus University Hospital, and Department of Clinical Medicine, Aarhus University, Aarhus, Denmark; 3 Department of Clinical Sciences, Lund University Diabetes Center, Lund University, Lund, Sweden; 4 Novo Nordisk Foundation Center for Basic Metabolic Research, University of Copenhagen, Copenhagen, Denmark; 5 Steno Diabetes Center Odense, Odense University Hospital, Odense, Denmark; 6 Steno Diabetes Center North Denmark, Aalborg University Hospital, Aalborg, Denmark; 7 Steno Diabetes Center Aarhus, Aarhus University Hospital, Aarhus, Denmark; 8 Department of Clinical Medicine, University of Copenhagen, Copenhagen, Denmark; 9 Department of Internal Medicine and Steno Diabetes Center Zealand, Holbæk Hospital, Holbæk, Denmark; 10 Department of Endocrinology, Skåne University Hospital, Malmö, Sweden

**Keywords:** Birthweight, Cardiovascular disease, Cohort study, Epidemiology, Fetal programming, Mortality, Stroke, Type 2 diabetes

## Abstract

**Aims/hypothesis:**

Low birthweight is a risk factor for type 2 diabetes and CVD. This prospective cohort study investigated whether lower birthweight increases CVD risk after diagnosis of type 2 diabetes.

**Methods:**

Original midwife records were evaluated for 8417 participants recently diagnosed with type 2 diabetes in the Danish Centre for Strategic Research in Type 2 Diabetes (DD2) cohort. Patients were followed for the first occurrence of a composite CVD endpoint (myocardial infarction, coronary revascularisation, peripheral arterial disease, stroke, unstable angina, heart failure or CVD death), a three-component endpoint comprising major adverse cardiovascular events (MACE), and all-cause mortality. Ten-year risks were estimated using the Aalen–Johansen estimator considering non-CVD death as a competing risk. HRs were determined by Cox regression. Models were controlled for sex, age, calendar year at birth, family history of diabetes and born-at-term status.

**Results:**

A total of 1187 composite CVD endpoints, 931 MACE, and 1094 deaths occurred during a median follow-up period of 8.5 years. The 10-year standardised composite CVD risk was 19.8% in participants with a birthweight <3000 g compared with 16.9% in participants with a birthweight of 3000–3700 g, yielding a risk difference (RD) of 2.9% (95% CI 0.4, 5.4) and an adjusted HR of 1.20 (95% CI 1.03, 1.40). The 10-year MACE risk for birthweight <3000 g was similarly elevated (RD 2.4%; 95% CI 0.1, 4.7; HR 1.22; 95% CI 1.01, 1.46). The elevated CVD risk was primarily driven by stroke, peripheral arterial disease and CVD death. All-cause mortality showed no substantial difference.

**Conclusions/interpretation:**

Having a birthweight <3000 g is associated with higher CVD risk among patients with type 2 diabetes, driven primarily by risk of stroke and CVD death.

**Graphical Abstract:**

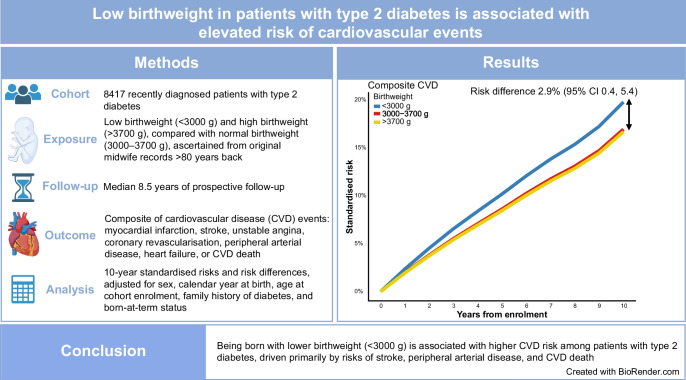

**Supplementary Information:**

The online version of this article (10.1007/s00125-024-06170-z) contains peer-reviewed but unedited supplementary material.


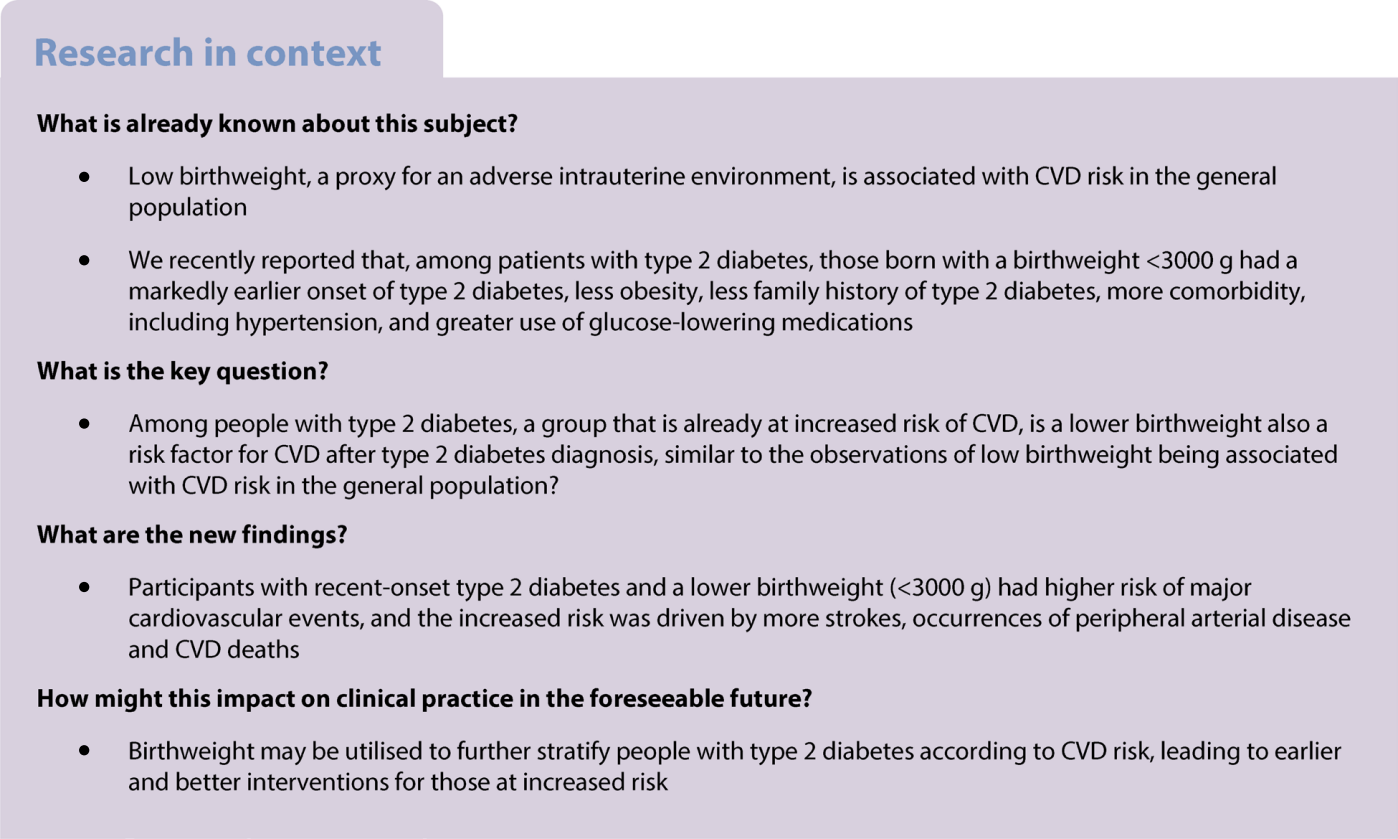


## Introduction

CVD remains the leading cause of morbidity and mortality in individuals with type 2 diabetes [1]. In the SCORE2-Diabetes algorithm [2], the main predictors of 10-year CVD risk in people with type 2 diabetes are established CVD risk factors, including age, smoking, systolic blood pressure and total and HDL-cholesterol, as well as diabetes-associated variables, including age at diabetes diagnosis, HbA_1c_ and estimated glomerular filtration rate [2].

Major risk factors for type 2 diabetes may be categorised into three groups: intrauterine environmental factors, postnatal environmental factors and genetic factors [3–5]. Key proxies for these three risk factor groups include birthweight, BMI in adulthood and type 2 diabetes polygenic risk scores [6]. Each factor appears to additively account for a majority of the total lifetime risk of type 2 diabetes [4]. The thrifty phenotype hypothesis provides a conceptual framework to help explain how exposure to an adverse fetal environment may cause multiple organ defects and dysfunctions, including changes in heart structure, dyslipidaemia, hypertension and atherosclerosis. When coupled with an affluent lifestyle, this may lead to overt type 2 diabetes and/or CVD [7, 8]. We recently reported that, among patients with type 2 diabetes, those with a birthweight <3000 g had markedly earlier onset of type 2 diabetes, less obesity and less family history of type 2 diabetes [3]. Lower birthweight among those recently diagnosed with type 2 diabetes was further associated with more comorbidity, including hypertension, and greater use of glucose-lowering medications [3]. These findings prompt questions of whether, among people with recently diagnosed type 2 diabetes, having a lower birthweight may also be a risk factor for CVD after type 2 diabetes diagnosis, similar to observations of low birthweight being associated with CVD risk in the general population [9]. Using a contemporary nationwide prospective cohort, we investigated the association of birthweight with subsequent risk of cardiovascular events and mortality in participants with recent-onset type 2 diabetes.

## Methods

### Study design and population

The Danish Centre for Strategic Research in Type 2 Diabetes (DD2) cohort is a Danish nationwide cohort of patients recently diagnosed with type 2 diabetes who have been enrolled by general practitioners and at hospital outpatient clinics since 2010 [10]. The enrolment process, implementation, logistics, biobanking and characteristics of the DD2 cohort have been described previously [10–12]. Briefly, clinical healthcare providers throughout Denmark identify individuals who have been recently diagnosed with type 2 diabetes and complete an online questionnaire, including items that require a physical examination. Urine and fasting blood samples are collected for storage in a biobank. The unique civil personal registration number assigned to all Danish citizens links participants in the DD2 cohort to a wide range of Danish health and administrative registries. Details regarding all data collected for the DD2 cohort are available in previous publications [10] and at www.dd2.dk. Information on baseline covariates, definitions and codes used in this study is provided in electronic supplementary material [ESM] Methods: DD2 cohort description and ESM Tables 1 and 2.

### Birthweight

The feasibility of extracting birthweight and associated variables for Danish residents in past decades through the Danish National Archives has been discussed previously [13]. Briefly, the Danish National Archives is a public institution that is responsible for collecting and storing historical documents from Denmark, including midwife records. The Danish midwife record system has a long history: since 1861, it has been mandatory by law for Danish midwives to keep records of all the deliveries and births in which they were involved [3]. For all participants in the DD2 cohort who were born between 1920 and 1988 and for whom data were available in the Danish National Archives, we retrieved information on birthweight, non-singleton birth status and born-at-term status that was objectively ascertained by midwives, as described previously [3]. For the birthweight of participants born after 1973 whose birth data could not be obtained from the midwife records, we retrieved date from the Danish Medical Birth Registry [14]. On the basis of our observations of dose–response relationships with CVD risk factors across the entire birthweight spectrum [3], we focused a priori on the lowest and highest birthweight quartiles [15]. Thus, we divided birthweight into birthweights below the lowest quartile (<25%, <3000 g) and above the highest quartile (>75%, >3700 g) the two middle quartiles (25–75%; 3000–3700 g) served as a reference group. We also analysed CVD outcomes when applying conventional clinical definitions of low birthweight (<2500 g) and high birthweight (>4500 g) [16].

### Outcomes

Outcomes were collected from the Danish National Patient Registry, which covers primary and secondary diagnosis codes and procedures for all inpatient hospital contacts in Denmark since 1977 and all outpatient hospital contacts since 1995 [17]. For most CVD events, e.g. myocardial infarction (MI) and stroke, we considered only inpatient discharge diagnoses because of their high predictive value [18, 19]. However, for heart failure (HF) and atrial fibrillation (Afib), we also included diagnoses from hospital specialist outpatient clinics and emergency department contacts without inpatient admission [20]. For all-cause mortality, exact dates of death were obtained from the Danish Civil Registration System. Information on the CVD death endpoint was obtained from a combination of the Danish National Patient Registry and the Danish Registry of Causes of Death. CVD death was assigned if CVD was listed as either an immediate or underlying cause of death on the death certificate, or if a primary or secondary inpatient diagnosis of CVD was followed by a record of death in the Danish Civil Registration System within 30 days (ESM Table 3).

The three main outcomes were a composite CVD endpoint, a three-component endpoint comprising major adverse cardiovascular events (MACE), and all-cause mortality. The composite CVD endpoint was defined as the first occurrence of MI, stroke, unstable angina pectoris, HF requiring hospitalisation, coronary revascularisation, peripheral arterial disease (PAD) or CVD death. MACE were defined as the first occurrence of MI and/or coronary revascularisation, stroke or CVD death.

Individual CVD events were defined as the first occurrence of MI, stroke, Afib, HF, PAD and CVD death. All diagnoses and procedure codes that were used are provided in ESM Table 3.

### Statistical analysis

Descriptive data are reported as medians and IQRs for continuous variables or counts and percentages for categorical variables for the three main exposure categories (birthweight <3000 g, 3000–3700 g, and >3700 g) and are tabulated by date of DD2 enrolment. For each CVD outcome, participants were followed from DD2 enrolment until the first occurrence of that outcome, death, emigration or the end of follow-up (1 June 2023), whichever came first. The median follow-up duration was calculated as the time from DD2 enrolment until death, emigration or the end of follow-up (1 June 2023). To account for missing data for covariates in the regression analysis, we performed multivariate imputations by chained equations using MICE package version 3.14.0 in R [21]. The percentage of missing values for covariates varied between 0% and 56%, with the highest percentage missing for blood pressure; the mean percentage of missing values across all covariates was 7%. The distribution of missingness was similar across birthweights, sexes and ages at enrolment. Predictive mean matching was used for imputation of continuous variables, logistic regression was used for binary variables and polytomous regression was used for categorical variables. Further details regarding the methods and missing data distributions are provided in ESM Methods: MICE model specification.

All analyses were performed using R statistical software version 4.1.2 (Vienna, Austria). We followed the STROBE (Strengthening the Reporting of Observational Studies in Epidemiology) guidelines.

#### Standardised risk

Using the Aalen–Johansen estimator, we calculated the standardised 10-year absolute risk estimates for our three main outcomes in the three birthweight categories, factoring in the competing risk of non-CVD death. We repeated the analyses for individual endpoints, accounting for the competing risk of death, except for CVD death, for which only non-CVD death was considered a competing event. For all-cause mortality, the Kaplan–Meier estimator was used. Next, to account for confounding, we used the parametric G-formula, which is similar to direct standardisation [22, 23]. We fitted two multivariable cause-specific Cox regression models, one to model the hazard rate for the outcome and one to model the hazard rate for the competing risk. Based on the two regression models and the G-formula [24], we calculated the absolute risk of an outcome according to birthweight category, and standardised it to the confounder distribution for the entire study population. Standardised risk differences (RDs) were estimated by comparing the <3000 g group and the >3700 g group with the reference birthweight group. Thus, the reported standardised absolute risks and RDs are the weighted averages of the conditional averages within each confounder stratum. We obtained 95% CIs through non-parametric bootstrapping with 10,000 samples [22]. All models were standardised for sex, age at DD2 enrolment, calendar year of birth, family history of type 2 diabetes and born-at-term status.

#### Hazard ratios

We calculated cause-specific HRs as a measure of the incidence rate ratio and 95% CIs using Cox proportional hazard models starting from the time of DD2 enrolment and comparing the groups with birthweight <3000 g and >3700 g with the reference birthweight group. Models were adjusted for potential confounders, i.e. sex, age and year of birth, family history of type 2 diabetes (sibling, parents or grandparents) and born-at-term status, identified by creating a directed acyclic graph (ESM Fig. 1). We deliberately avoided further extensive multivariable adjustments in our main model because the aim was to compare CVD risk in participants with type 2 diabetes according to birthweight category, with birthweight being an exposure that is already defined at birth. Socio-behavioural, metabolic and lifestyle factors measured later in life may act as intermediate factors between birthweight, type 2 diabetes and later CVD outcomes of interest. To further examine whether any association of birthweight with CVD events was independent of potential intermediate factors, we performed additional exploratory analyses adjusted for behavioural lifestyle factors (including physical activity, smoking status and alcohol consumption), socioeconomic markers (including marital status and level of urbanisation) and BMI, as well as the number of antihypertensive medications (as a proxy for hypertension burden) and the number of glucose-lowering medications (as a proxy for type 2 diabetes dysmetabolic state). The proportional hazards assumption in the models was fulfilled according to Schoenfeld or Martingale residual plots after inclusion of age at enrolment as a categorical variable.

Finally, in a Cox regression analysis, we modelled birthweight as a continuous predictor using linear, polynomial and restricted cubic spline functions [25, 26] to explore the relationship between continuous birthweight and the adjusted HR (aHR) of the primary outcomes. A median birthweight of 3400 g was used as the reference (HR=1) in these analyses. Spline models were evaluated through visual inspection, likelihood ratio tests (*p* value <0.05) and Akaike’s information criterion.

#### Sensitivity analysis

To assess any effect of birthweight on CVD risk in participants without prior CVD at type 2 diabetes diagnosis, data were re-analysed after excluding participants with a CVD hospital contact within 10 years prior to enrolment. Because age at type 2 diabetes onset may be an effect of birthweight, we also re-ran our Cox regression models without including age at DD2 enrolment as a covariate. To explore associations in specific subgroups, we performed analyses stratified by sex, analyses restricted to those born at term and analyses stratified by calendar year at birth. Finally, we calculated sub-distributional aHR using the Fine–Gray model.

### Ethics

This study was approved by the Danish Data Protection Agency (record number 2008-58-0035) and the Regional Committees on Health Research Ethics for Southern Denmark (record number S-20100082). All participants provided written informed consent to participate in the DD2 study.

## Results

The DD2 cohort enrolled 11,375 patients in the period 2010–2023. A total of 2686 participants were born after 1988, had an unknown birthplace or had incomplete birth data. We then excluded 130 patients with non-singleton births and 142 who were positive for GAD antibody (>30 kU/l) (to avoid potential misclassification with autoimmune diabetes). The final study population included 8417 participants with recently diagnosed type 2 diabetes (ESM Fig. 2). A total of 2152 participants (25.6%) had a birthweight <3000 g, 4262 (50.6%) had a birthweight of 3000–3700 g and 2003 (23.8%) had a birthweight >3700 g. Table 1 shows the overall baseline characteristics according to birthweight category. As reported previously [3], the median age at enrolment was 61 years, and participants with birthweight <3000 g were 6 years younger at type 2 diabetes onset than those with birthweight >3700 g. Birthweight <3000 g was associated with female sex, fewer relatives with type 2 diabetes and a lower BMI (in particular, a lower likelihood of being in the groups with BMI >35 kg/m^2^; Table 1). Birthweight <3000 g was also associated with less use of antihypertensive medication and greater use of glucose-lowering medication than participants with a birthweight of 3000–3700 g. Further detailed information on covariates is provided in ESM Tables 4–6.

**Table 1:** Baseline characteristics at enrolment, according to birthweight

Variable	<3000 g (*n*=2152)	3000–3700 g (*n*=4262)	>3700 g (*n*=2003)	Total (*n*=8417)
Sex				
Male	1064 (49.4)	2528 (59.3)	1364 (68.1)	4956 (58.9)
Female	1088 (50.6)	1734 (40.7)	639 (31.9)	3461 (41.1)
Age at enrolment (years)				
Median (IQR)	57.8 (49.2, 65.8)	61.5 (52.4, 68.2)	63.9 (54.3, 69.8)	61.1 (51.9, 68.1)
<45	297 (13.8)	441 (10.3)	174 (8.7)	912 (10.8)
45–55	612 (28.4)	907 (21.3)	364 (18.2)	1883 (22.4)
55–65	651 (30.3)	1323 (31.0)	544 (27.2)	2518 (29.9)
65–75	479 (22.3)	1262 (29.6)	714 (35.6)	2455 (29.2)
>75	113 (5.3)	329 (7.7)	207 (10.3)	649 (7.7)
Family history of type 2 diabetes (number of relatives)				
0	1063 (49.4)	2087 (49.0)	913 (45.6)	4063 (48.3)
1	666 (30.9)	1316 (30.9)	660 (33.0)	2642 (31.4)
2	320 (14.9)	629 (14.8)	313 (15.6)	1262 (15.0)
3+	103 (4.8)	230 (5.4)	117 (5.8)	450 (5.3)
Born-at-term status				
Term	1324 (61.5)	4060 (95.3)	1927 (96.2)	7311 (86.9)
Preterm	828 (38.5)	202 (4.7)	76 (3.8)	1106 (13.1)
BMI (kg/m^2^)				
Median (IQR)	31.1 (27.4, 34.9)	31.1 (27.5, 35.8)	31.6 (28.1, 36.1)	31.2 (27.6, 35.7)
<25	144 (12.2)	247 (10.7)	104 (9.6)	495 (10.8)
25–30	362 (30.6)	722 (31.4)	320 (29.5)	1404 (30.7)
30–35	386 (32.6)	668 (29.1)	323 (29.8)	1377 (30.2)
35–40	186 (15.7)	370 (16.1)	200 (18.4)	756 (16.6)
>40	105 (8.9)	292 (12.7)	138 (12.7)	535 (11.7)
Missing	969	1963	918	3850
Waist circumference (cm)				
Median (IQR)	107 (100, 116)	108 (101, 117)	110 (103, 118)	108 (101, 117)
Missing	40	58	34	132
Alcohol consumption				
>14/21 units per week (male/female)	99 (4.6)	291 (6.8)	123 (6.2)	513 (6.1)
Missing	<5	<5	<5	11
Smoking status				
Never	491 (50.5)	953 (48.3)	428 (44.9)	1872 (48.0)
Former	282 (29.0)	678 (34.3)	331 (34.7)	1291 (33.1)
Current	199 (20.5)	344 (17.4)	194 (20.4)	737 (18.9)
Missing	1180	2287	1050	4517
Physical activity (days per week)				
0	293 (13.6)	615 (14.4)	283 (14.1)	1191 (14.1)
1 or 2	474 (22.0)	872 (20.5)	402 (20.1)	1748 (20.8)
3 or 4	503 (23.4)	991 (23.3)	468 (23.4)	1962 (23.3)
5 or 6	349 (16.2)	693 (16.3)	296 (14.8)	1338 (15.9)
7	531 (24.7)	1090 (25.6)	554 (27.7)	2175 (25.8)
Marital status				
Married/partnership	1250 (60.2)	2535 (61.4)	1242 (63.8)	5027 (61.7)
Divorced/separated	351 (16.9)	679 (16.4)	280 (14.4)	1310 (16.1)
Non-married/no registered partnership	339 (16.3)	601 (14.6)	242 (12.4)	1182 (14.5)
Widow/widower	137 (6.6)	314 (7.6)	182 (9.4)	633 (7.8)
Missing	75	133	57	265
Level of urbanisation				
Capital municipalities	332 (15.4)	634 (14.9)	271 (13.5)	1237 (14.7)
Large city municipalities	442 (20.5	943 (22.1)	400 (20.0)	1785 (21.2)
Provincial municipalities	563 (26.2)	1062 (24.9)	527 (26.3)	2152 (25.6)
Surrounding area municipalities	455 (21.1)	882 (20.7)	443 (22.1)	1780 (21.2)
Rural area municipalities	359 (16.7)	741 (17.4)	362 (18.1)	1462 (17.4)
Missing	<5	<5	<5	<5
SBP (mmHg)				
Median (IQR)	130 (125, 140)	131 (125, 140)	130 (124, 140)	130 (125, 140)
Missing	1201	2329	1068	4598
DBP				
Median (IQR)	80 (75, 87)	80 (75, 86)	80 (74.5, 86.0)	80 (75, 86)
Missing	1201	2329	1068	4598
Resting heart rate (bpm)				
Median (IQR)	70 (64, 80)	70 (63, 78)	69 (63, 78)	70 (64, 79)
Missing	38	47	28	113
Total cholesterol (mmol/l)				
Median (IQR)	4.3 (3.7, 5.1)	4.3 (3.7, 5.1)	4.3 (3.6, 5.0)	4.3 (3.7, 5.1)
Missing	619	1257	595	2471
Triglycerides (mmol/l)				
Median (IQR)	1.8 (1.3, 2.6)	1.8 (1.2, 2.5)	1.7 (1.2, 2.4)	1.7 (1.2, 2.5)
Missing	499	1017	488	2004
HDL-cholesterol (mmol/l)				
Median (IQR)	1.1 (1.0, 1.4)	1.1 (1.0, 1.4)	1.1 (1.0, 1.4)	1.1 (1.0, 1.4)
Missing	625	1261	597	2483
LDL-cholesterol (mmol/l)				
Median (IQR)	2.2 (1.7, 2.9)	2.3 (1.7, 2.9)	2.2 (1.7, 2.9)	2.2 (1.7, 2.9)
Missing	495	1009	482	1986
Blood glucose (mmol/l)				
Median (IQR)	7.2 (6.4, 8.3)	7.2 (6.4, 8.2)	7.1 (6.4, 8.2)	7.2 (6.4, 8.3)
Missing	1051	1962	925	3938
HbA_1c_				
Median (IQR), mmol/mol	48.0 (44.0, 55.1)	48.0 (44.0, 54.1)	48.0 (44.0, 55.1)	48.0 (44.0, 55.1)
Median (IQR), %	6.5 (6.2, 7.2)	6.5 (6.2, 7.1)	6.5 (6.2, 7.2)	6.5 (6.2, 7.2)
Missing	300	602	295	1197
C-peptide (pmol/l)				
Median (IQR)	1145.5 (877.2, 1559.8)	1176.0 (864.7, 1557.0)	1197.0 (889.9, 1647.5)	1172.0 (876.2, 1576.0)
Missing	978	1767	835	3580
HOMA2-B				
Median (IQR)	92.6 (69.6, 118.8)	92.6 (70.1, 119.5)	96.1 (71.5, 125.7)	93.4 (70.3, 121.4)
Missing	1061	1982	931	3974
HOMA2-insulin sensitivity				
Median (IQR)	35.2 (25.9, 47.1)	34.5 (25.7, 47.4)	33.4 (24.3, 46.0)	34.6 (25.5, 47.0)
Missing	1061	1982	931	3974
HOMA2-IR				
Median (IQR)	2.8 (2.1, 3.9)	2.9 (2.1, 3.9)	3 (2.2, 4.1)	2.9 (2.1, 3.9)
Missing	1061	1982	931	3974
hsCRP (mg/l)				
Median (IQR)	2.2 (0.9, 4.7)	2.0 (0.9, 4.6)	2.0 (0.8, 4.2)	2.0 (0.9, 4.6)
Missing	623	1114	510	2247

Values are *n* (%) unless otherwise indicated

DBP, diastolic blood pressure; hsCRP, high-sensitivity C-reactive protein; SBP, systolic blood pressure

### Main outcomes: composite CVD, MACE and all-cause mortality

**Fig. 1:**
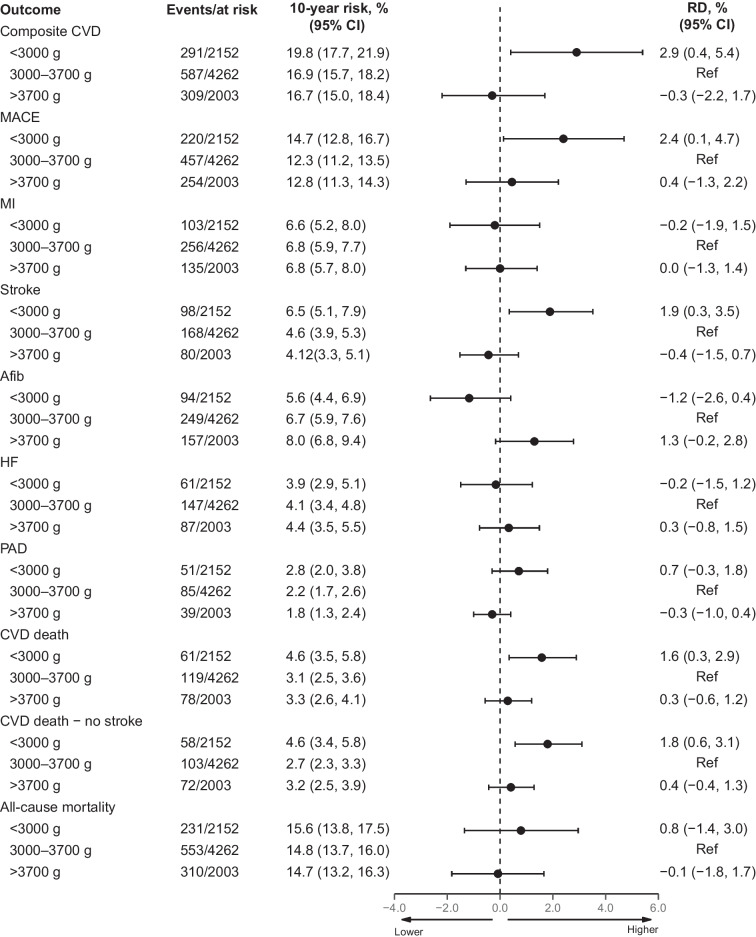
Ten-year standardised risk and RD for cardiovascular endpoints according to birthweight. The Forest plot is standardised according to the distribution of sex, age at DD2 enrolment, calendar year of birth, family history of type 2 diabetes and born-at-term status. The composite CVD endpoint includes MI, stroke, unstable angina pectoris, coronary revascularisation, PAD, HF requiring hospitalisation or CVD death. MACE include MI and coronary revascularisation, stroke or CVD death

**Fig. 2:**
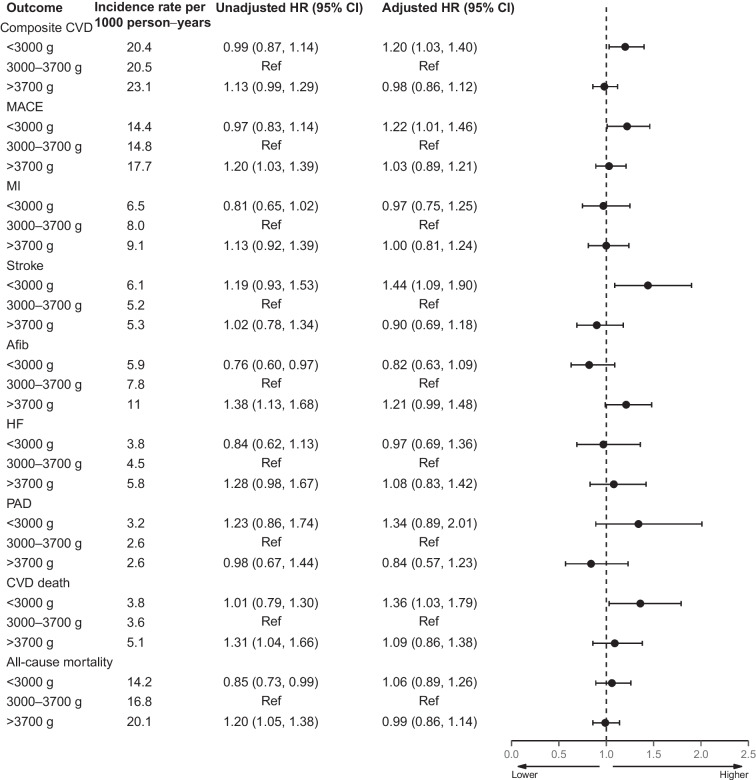
HRs for cardiovascular endpoints according to birthweight. The reference birthweight group (3000–3700 g) was compared with the lower birthweight category (<3000 g) and the higher birthweight category (>3700 g), adjusted for sex, age at DD2 enrolment, calendar year of birth, family history of type 2 diabetes and born-at-term status. The composite CVD endpoint includes MI, stroke, unstable angina pectoris, coronary revascularisation, PAD, HF requiring hospitalisation or CVD death. MACE include MI and coronary revascularisation, stroke or CVD death

**Fig. 3:**
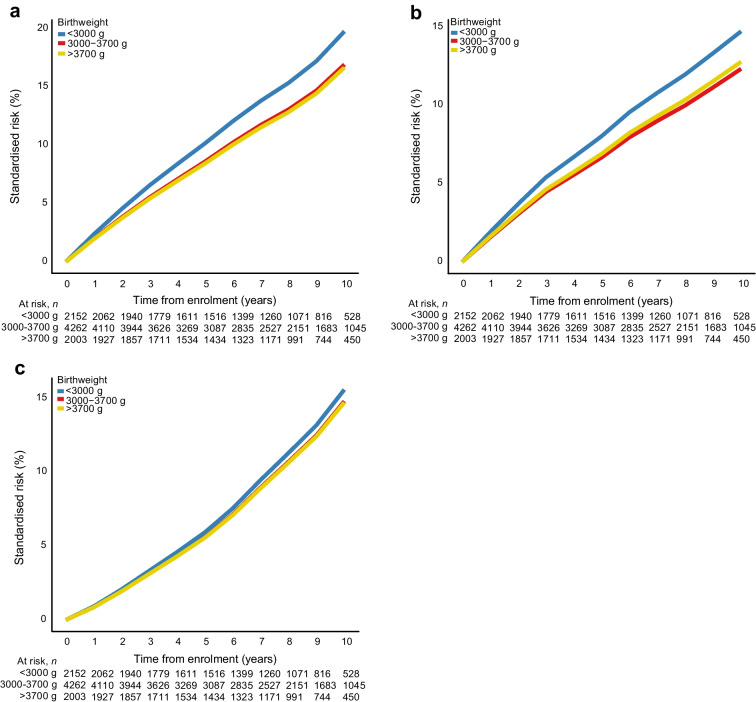
Standardised risk curves according to birthweight. (**a**) Results for the main composite CVD endpoint after DD2 enrolment, standardised to the distribution of sex, age at DD2 enrolment, calendar year of birth, family history of type 2 diabetes and born-at-term status. The composite CVD endpoint includes MI, stroke, unstable angina pectoris, coronary revascularisation, PAD, HF requiring hospitalisation or CVD death. (**b**) Results for the main MACE endpoint after DD2 enrolment, standardised to the distribution of sex, age at DD2 enrolment, calendar year of birth, family history of type 2 diabetes and born-at-term status. MACE include MI and coronary revascularisation, stroke or CVD death. (**c**) Results for all-cause mortality after DD2 enrolment, standardised to the distribution of sex, age at DD2 enrolment, calendar year of birth, family history of type 2 diabetes, and born-at-term status. Blue lines, low birthweight group; red lines, reference group; yellow lines, high birthweight group

A total of 1187 composite CVD endpoints, 931 MACE and 1094 deaths occurred (ESM Table 5) during an overall median follow-up period of 8.5 years (IQR 5.4, 10.1). The 10-year standardised risk of a composite CVD endpoint was highest in participants with a birthweight <3000 g, 19.8% compared with 16.9% in participants with a birthweight of 3000–3700 g, corresponding to a 10-year standardised RD of 2.9% (95% CI 0.4, 5.4) and an aHR of 1.20 (95% CI 1.03, 1.40; Figs. 1, 2 and 3a). For MACE, the 10-year standardised risk was similarly higher, 14.7% in participants with a birthweight <3000 g vs 12.3% among participants with a birthweight of 3000–3700 g, corresponding to a 10-year standardised RD of 2.4% (95% CI 0.1, 4.7) and an aHR of 1.22 (95% CI 1.01, 1.46; Figs. 1, 2 and 3b). The 10-year standardised risk of all-cause mortality was more similar between groups, being 15.6% in participants with a birthweight <3000 g vs 14.8% in participants with a birthweight of 3000–3700 g, with a standardised RD of 0.8% (95% CI −1.4, 3.0) and an aHR of 1.06 (95% CI 0.89, 1.26; Figs. 1, 2 and 3c).

The 10-year standardised composite CVD endpoint risk was similar for the group with birthweight >3700 g and the group with birthweight 3000–3700 g (16.7% vs 16.9%, respectively), with an aHR of 0.98 (95% CI 0.86, 1.12) (Figs. 1, 2 and 3a). For MACE, the 10-year standardised risk was 12.8% in participants with a birthweight >3700 g vs 12.3% in participants with a birthweight of 3000–3700 g, and the aHR was 1.03 (95% CI 0.89, 1.21 Figs. 1, 2 and 3b). The 10-year standardised risk of all-cause mortality was 14.7% in participants with a birthweight >3700 g vs 14.8% in participants with a birthweight of 3000–3700 g, and the aHR was 0.99 (95% CI 0.86, 1.14; Figs. 1, 2 and 3c).

ESM Table 7 shows the results from stepwise confounder adjustments. Notably, the crude hazards/risks for CVD were not higher in participants with birthweight <3000 g (aHR 0.99; 95% CI 0.87, 1.14) but were higher in those with birthweight >3700 g (aHR 1.13; 95% CI 0.99, 1.29) compared with the normal birthweight group. After adjusting for the association of female sex and younger age with a birthweight <3000 g, the aHRs increased to 1.20 (95% CI 1.03, 1.40) for a birthweight <3000 g and decreased to an aHR of 0.98 (95% CI 0.86, 1.12) for a birthweight >3700 g in the main model. Further adjustments for alcohol consumption, smoking status, physical activity, marital status, level of urbanisation, BMI and the number of glucose-lowering and antihypertensive medications showed similar risk associations (ESM Table 7).

### Individual cardiovascular endpoints

Compared with a birthweight of 3000–3700 g, a birthweight <3000 g was not associated with greater risk of MI, Afib or HF (Figs. 1 and 2). However, a birthweight <3000 g was associated with a greater risk of stroke, with a 10-year standardised risk of 6.5% vs 4.6%, corresponding to a 10-year standardised RD of 1.9% (95% CI 0.3, 3.5) and an aHR of 1.44 (95% CI 1.09, 1.90). A birthweight <3000 g was also associated with greater risk of CVD death vs a birthweight of 3000–3700 g, with a 10-year standardised risk of 4.6% vs 3.1%, corresponding to a 10-year standardised RD of 1.6% (95% CI 0.3, 2.9) and an aHR of 1.36 (95% CI 1.03, 1.79). Notably, removal of all stroke-associated deaths from the CVD death endpoint did not attenuate the elevated risk of CVD death (Fig. 1 and ESM Table 7). A birthweight <3000 g was also associated with an elevated risk of PAD, although with limited statistical precision (related to fewer events), with a 10-year standardised risk of 2.8% vs 2.2%, corresponding to a 10-year standardised RD of 0.7% (95% CI −0.3, 1.8) and an aHR of 1.34 (95% CI 0.89, 2.01).

For participants with a birthweight >3700 g, no associations with greater risk of MI, stroke, HF, CVD death or PAD were observed vs those with a birthweight of 3000–3700 g (Figs. 1 and 2). A birthweight >3700 g yielded a greater, although imprecise, 10-year standardised risk of Afib vs a birthweight of 3000–3700 g (8.0% vs 6.7%, respectively), corresponding to a 10-year standardised RD of 1.3% (95% CI −0.2, 2.8) and an aHR of 1.21 (95% CI 0.99, 1.48). Further adjustments for alcohol consumption, smoking status, physical activity, marital status, level of urbanisation, BMI and the number of glucose-lowering and antihypertensive medications did not materially change the estimates (ESM Table 7).

### Continuous birthweight analyses

According to likelihood ratio tests, no models showed a difference with a *p* value <0.05 from the linear regression models, and the Akaike’s information criterion values were all generally similar. Visual inspection indicated that the models became overfitted when the restricted cubic spline models contained more than four knots. For the composite CVD and MACE endpoints, all models showed similar patterns of a successively increasing aHR with gradually lower birthweight with respect to the median birthweight of 3400 g. For all-cause mortality, most models were uniform without evidence of any material difference in mortality risk by birthweight. However, with six or more knots, we observed a tendency towards slightly decreasing all-cause mortality with higher birthweight (ESM methods: Exploring birthweight as a continuous exposure using restricted cubic spline regression).

### Sensitivity analysis

Re-analysis of our data with application of clinically defined low (<2500 g) and high (>4500 g) birthweight yielded results comparable to those for the <3000 g and >3700 g birthweight groups, with a tendency toward larger estimates with less statistical precision (ESM Table 8). For individual CVD endpoints, the numbers of events in the clinically defined birthweight groups were too small to yield reliable results (10, 17 and 13 events for MI, stroke, and CVD death, respectively, in the <2500 g birthweight group).

When restricting analyses to participants with type 2 diabetes without pre-existing CVD, the 10-year standardised risks of CVD endpoints and death were, as expected, lower than those in the total type 2 diabetes cohort (ESM Table 9 and ESM Fig. 3). The risk estimates for associations of composite CVD and MACE with birthweights <3000 g were lower than those in the main analyses (ESM Table 9 and ESM Fig. 3).

The Fine–Gray sub-distribution models showed similar associations to those for the cause-specific Cox models (ESM Table 10). In sex-stratified analyses, the risk patterns differed between sexes. Birthweight was divided into the lowest quartile and above the highest quartile, using the two middle quartiles as a reference for female and male participants individually. For female participants, the aHR increases for composite CVD and MACE in those with a birthweight <3000 g compared with 3000–3600 g was less pronounced (CVD 1.10; 95% 0.84, 1.44; MACE 1.00; 95% CI 0.73, 1.37) compared with male participants (CVD 1.25; 95% 1.06, 1.48; MACE 1.31; 95% CI 1.09, 1.58). This effect modification was even more pronounced for the risk of stroke in female participants with birthweight <3000 g (1.00; 95% CI 0.62, 1.62) vs male participants with birthweight <3100 g (1.55; 95% CI 1.13, 2.12) (ESM Table 11). For male participants, a birthweight <3100 g, compared with a birthweight 3100–3750 g, was associated with an increased aHR for MI (1.21; 95 % CI 0.93, 1.57), which was not observed for women with a birthweight <3000 g (0.81; 95% CI 0.49, 1.36) (ESM Table 11). However, statistical precision in the sex-stratified analyses was limited. Continuous birthweight models for female participants only, assessing composite CVD and MACE endpoints, revealed a pattern of increased aHR with decreasing birthweight, and this became more pronounced in spline models (ESM methods: Exploring sex stratified analysis with birthweight as a continuous exposure suing restricted cubic spline regression). Conversely, continuous birthweight models for male participants mirrored the patterns of the non-stratified analysis (ESM methods: Exploring sex stratified analysis with birthweight as a continuous exposure suing restricted cubic spline regression).

Stratified analysis by calendar year at birth indicated an increased aHR for all-cause mortality for participants born after 1953, both for those with a birthweight <3000 g (although with limited statistical precision) and for those with a birthweight >3700 g. Thus, compared with the reference group (birthweight 3000–3700 g), all-cause mortality aHRs were 1.27 (95% CI 0.88, 1.83) for birthweight <3000 g and 1.45 (95% CI 1.03, 2.04) for birthweight >3700 g. However, a clearly increased CVD mortality aHR for a birthweight <3000 g was only observed for participants born from 1920–1953 (ESM Table 11). Restricting the analysis to those born at term yielded similar estimates to the non-stratified analysis (ESM Table 11).

## Discussion

In this prospective study of 8417 participants with recently diagnosed type 2 diabetes, a birthweight <3000 g was associated with elevated CVD risk, primarily because of increased risks of stroke and CVD death. We are not aware of any previous large-scale studies investigating the association between low birthweight and CVD outcomes among patients with new-onset type 2 diabetes. This is clinically important because this group is already enriched with people with low birthweight, which is a risk factor for type 2 diabetes development [3], and has an inherent increased CVD risk compared with the general population [1].

In contrast to a previous study of only 171 participants with type 2 diabetes [27], we found no clear association with all-cause mortality, but observed an elevated risk of CVD death in participants with a birthweight <3000 g. The association remained robust after exclusion of stroke-associated deaths, and may indicate greater severity of events and a lower survival rate after major cardiovascular events among people with a lower birthweight. Previous findings in populations that included individuals with and without diabetes [28, 29] have shown a consistently increased risk of CVD death associated with a low birthweight, and some studies [29] have also indicated a higher risk of all-cause mortality. Considering the median age of 61 years in the DD2 cohort and the fact that inclusion in this cohort requires survival until the onset of type 2 diabetes, cohort participants with a low birthweight may represent the healthiest and most resilient people within their generation. This would probably lead us to underestimate any impact of low birthweight on CVD risk in type 2 diabetes patients.

Young age at type 2 diabetes onset has recently been recognised as a risk factor for CVD in type 2 diabetes, and is included in the SCORE2-Diabetes algorithm [2]. Given the pronounced effects of lower birthweight on age at type 2 diabetes onset [3] and diagnosis [4], a substantial proportion of the predictive value of adding age at type 2 diabetes onset to the SCORE2 algorithm may capture the effects of an adverse fetal environment, as reflected by a lower birthweight.

Our finding that much of the elevated CVD risk in participants with a birthweight <3000 g was driven by an elevated risk of stroke is consistent with findings from a recent study of self-reported birthweight and CVD outcomes in a population with or without diabetes from the UK Biobank [9]. Low birthweight has consistently been associated with hypertension, even at relatively young ages, in people with or without diabetes [3, 15, 30–32]. Hypertension is a dominant risk factor for stroke [33] compared with other CVD outcomes, and may be an important mediator of the elevated stroke risk among patients with type 2 diabetes with the lowest birthweights. Interestingly, we also found that PAD was associated with lower birthweight. However, because of the low number of events, these results were imprecise, with parameter values ranging from no effect to a substantial increase in risk. In contrast to our findings, Liang et al reported greater risk of HF in individuals with lower birthweight, potentially due to the inclusion of individuals without diabetes [9]. In addition to hypertension and type 2 diabetes, lower birthweight has been found to be associated with other CVD risk factors, such as dyslipidaemia, abdominal obesity, insulin resistance and elevated liver fat [34–37], possibly affecting our results.

We found no association with greater risk of CVD for participants with the highest 25% of birthweights, which is consistent with the results of the UK Biobank study [9]. Interestingly, we observed a greater risk of Afib in participants with the highest 25% of birthweights, although this finding had low statistical precision. Given that obesity is a strong risk factor for Afib [38], this may potentially be related to their higher BMI at the onset of type 2 diabetes [3].

Stratified analyses revealed a stronger relationship between lower birthweight and the risk of CVD death and stroke in men compared with women. Whether this association is related to higher age-adjusted incidence rates of stroke in men is unknown and requires further studies [39]. Likewise, further studies are needed to determine the extent to which the differential associations between birthweight and all-course mortality vs CVD death among patients born before vs after 1953 reflect true birth-period effects.

The strengths of this large nationwide study include the use of a well-characterised cohort of participants recently diagnosed with type 2 diabetes, recruited from both the primary and secondary healthcare sectors, with linkage to data from high-quality population-based health registries. In addition, birthweight was independently ascertained from original midwife records spanning almost 100 years, preventing recall bias.

The limitations of this study include the potential for survival and/or selection bias before entering the DD2 cohort. Both situations would probably have decreased participation among individuals with high cardiovascular risk or low birthweight, and therefore most likely biased results toward the null hypothesis, underestimating CVD rates among individuals with low birthweight and type 2 diabetes. Furthermore, low birthweight is a risk factor for common lung, mental and neurological diseases, which may occur earlier in life than type 2 diabetes and CVD [15, 35, 36, 40–43]. The relevance and/or influence of this on our current results are unknown. We generally lacked information on factors present between birth and adulthood in our participants, such as early socio-behavioural factors, education, occupation and income, which may act as mediators of any impact of low birthweight on their later type 2 diabetes and CVD risk but may also alternatively reflect unmeasured confounders among parents leading to both lower birthweight and later type 2 diabetes and CVD in offspring. The validity of CVD diagnoses and procedures in Danish registries is very high [19, 20], and thus misclassification of diagnoses probably did not adversely influence the results. Data were missing for some covariates. However, the variables included in our main model had relatively few missing data, and we used multiple imputations to account for missing data when using regression methods. All individuals were born in Denmark, and thus our study population was likely homogeneous in terms of race/ethnicity although this was not specifically addressed. Further studies of different ethnicities and races are needed. Finally, low birthweight may not be the causal factor per se for increased CVD risk in type 2 diabetes, but a marker of many different fetal exposures, including the mother’s nutritional and general health status, smoking, medications, stress and socioeconomic and/or psychological determinants, which may have adversely affected long-term organ structures and/or functions [31]. Nevertheless, the fact that all of these factors influence fetal growth suggests that birthweight may be considered a marker of multiple early-life programming causes of later CVD risk in type 2 diabetes.

In conclusion, our prospective study reveals an association between lower birthweight and an elevated risk of CVD among patients with type 2 diabetes, a group who are already at increased baseline risk for CVD. Further studies are needed to determine the extent to which birthweight could be integrated into CVD risk assessments for people with type 2 diabetes.

### Supplementary Information

Below is the link to the electronic supplementary material.

ESM (PDF 2166 KB)

## Data Availability

Danish data protection legislation does not allow the authors to share any individual-level patient data. However, the Danish health registry data used in this study are accessible to researchers at authorised research institutions by application to the Danish Health Data Authority by email (forskerservice@sundhedsdata.dk). Requests to use data from the DD2 cohort can be made at the DD2 website (https://dd2.dk/forskning/ansoeg-om-data)

## Abbreviations

Afib: Atrial fibrillation
aHR: Adjusted hazard ratio
DD2: Danish Centre for Strategic Research in Type 2 Diabetes cohort
HF: Heart failure
MACE: Major adverse cardiovascular events
MI: Myocardial infarction
PAD: Peripheral arterial disease
RD: Risk difference
