# Supplementary material for: Low birthweight in patients with type 2 diabetes is associated with elevated risk of cardiovascular events and mortality
Source: Diabetologia. 2024 May 22;67(8):1616–29. doi: 10.1007/s00125-024-06170-z (PMC11343788; doi:10.1007/s00125-024-06170-z)
Supplement: Supplementary file 1 — ESM (PDF 2166 KB) [file 125_2024_6170_MOESM1_ESM.pdf]

Electronic supplementary material

Table of contents

Electronic supplementary material 1
ESM methods: 2
DD2 Cohort description 2
Multivariate Imputations by Chained Equations (MICE) model specification 3
Exploring birthweight as a continuous exposure using restricted cubic spline regression. 7
Exploring sex stratified analysis with birthweight as a continuous exposure using restricted cubic spline regression. 11
ESM Tables 14
ESM table 1: data sources 14
ESM Table 2: Variable definitions 15
ESM Table 3: Outcome definitions 22
ESM Table 4: Medication according to birthweight 24
ESM table 5: Incident cardiovascular endpoints according to birthweight 25
ESM Table 6: Cardiovascular endpoints and comorbidities, prior to DD2 enrolment, according to birthweight 26
ESM Table 7: Stepwise adjustments in cox proportional hazard regression of cardiovascular endpoints according to birthweight 27
ESM Table 8: Stepwise adjustments in cox proportional hazard regression models of cardiovascular endpoints according to conventional clinically defined birthweights. 30
ESM Table 9: Stepwise adjustments in cox proportional hazard regression models of cardiovascular endpoints according to birthweight without any pre-existing cardiovascular disease. 32
ESM Table 10: Stepwise adjustments in Fine and Gray sub-distributional models of cardiovascular endpoints according to birthweight 34
ESM Table 11: Cardiovascular endpoints according to birthweight: Results stratified by sex, restricted to individuals born at term, and stratified by calendar year of birth, respectively 36
ESM Figures 38
ESM Fig. 1: Directed acyclic graph 38
ESM Fig. 2: Flowchart of the study population 39
ESM Fig. 3: Ten-year standardized risk and risk differences for cardiovascular endpoints according to birthweight, without pre-existing cardiovascular disease 40
ESM references 41

ESM methods:

DD2 Cohort description

The Danish Centre for Strategic Research in Type 2 Diabetes (DD2) cohort

Study participants and recruitment process of the DD2 cohort have been thoroughly documented by Christensen et al[1]. Since its inception on 1 January 2009, all patients with newly or recently diagnosed type 2 diabetes in Denmark have been eligible to participate in the DD2 cohort, enrolling its first participant in November 2010. As of 2024, the cohort includes 11375 participants, with the cohort continuing to enrol new participants. The enrolment for the DD2 cohort is structured as follows: clinical providers, typically general practitioners, or hospital nurses, identify patients newly diagnosed with type 2 diabetes during routine clinical care. These patients are then informed about the DD2 project and provided with both oral and written details regarding participation. Interested patients sign a written informed consent form, after which their clinical information is collected through an online questionnaire completed by the clinical provider, which includes items requiring a physical examination. Furthermore, urine and fasting blood samples are collected for biobank storage. Diagnosis of type 2 diabetes has adhered to WHO criteria. Before 2012 primarily based on Oral Glucose Tolerance Test (OGTT), and subsequently, from 2012 onwards, on a glycosylated haemoglobin A ($HbA1_c$) >48 mmol/mol (6.5%). While the DD2 initially aimed to focus on newly diagnosed patients with type 2 diabetes, practicalities of clinical practice mean that referrals to the DD2 may not occur at once upon diabetes diagnosis when other clinical concerns take precedence. Therefore, individuals diagnosed with type 2 diabetes sometime after 2009 but before their DD2 enrolment were accepted. While we do not have exact date of diabetes diagnosis for all DD2 participants, mean duration from the first glucose-lowering medication initiation to DD2 enrolment is 1 to 1.5 years. The exact coverage of all incident type 2 diabetes in Denmark by the DD2 cohort is unknown. However, with an annual enrolment rate of 1000-1200 participants, the DD2 enrols an estimated 5% of all people with newly diagnosed type 2 diabetes nationwide. Recruitment across Denmark's five healthcare regions varies, with the largest segment (35%) of the cohort being recruited from the region of Southern Denmark, which represents 21% of the national population. This is followed by the Central Denmark Region and the Capital Region, contribution 24% and 19% of the cohort participants, respectively, against their population shares of 22% and 31%. Participant attrition in the DD2 cohort occurs due to emigration or death, events which are tracked through linkage with the Danish Civil Registration System. All enrolled patients retain the right to withdraw from the DD2 cohort at any time.

Multivariate Imputations by Chained Equations (MICE) model specification

We employed multivariate imputations by chained equations using the MICE package from R[2]. The percentage of missing values across the dataset ranged from 0 to 56%, with an average of 7% missing values across all variables. Included below are plots showing the percentage of missing data per variable according to birthweight, age at enrolment, and sex. The plots show that the distributions of missing data according to birthweight categories, age at enrolment, and sex were similar, providing evidence against potential attrition bias.

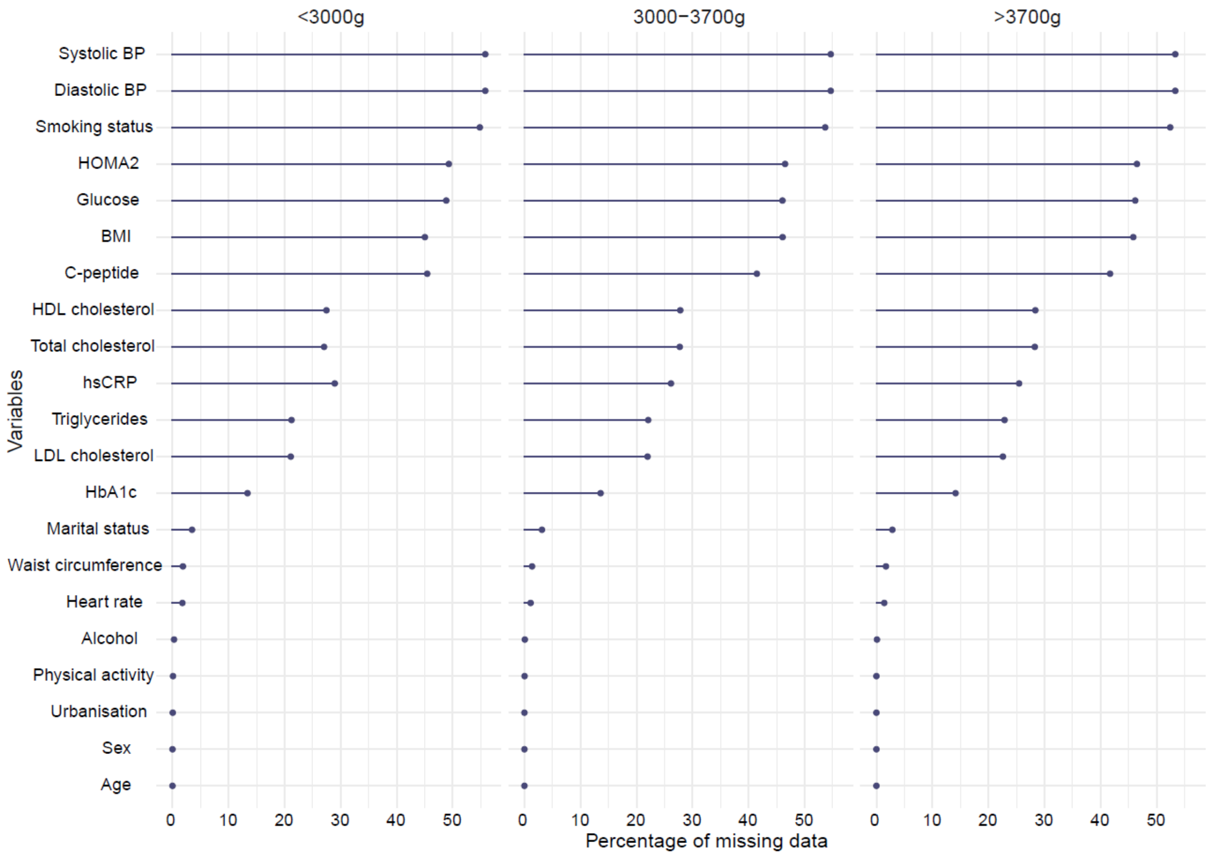

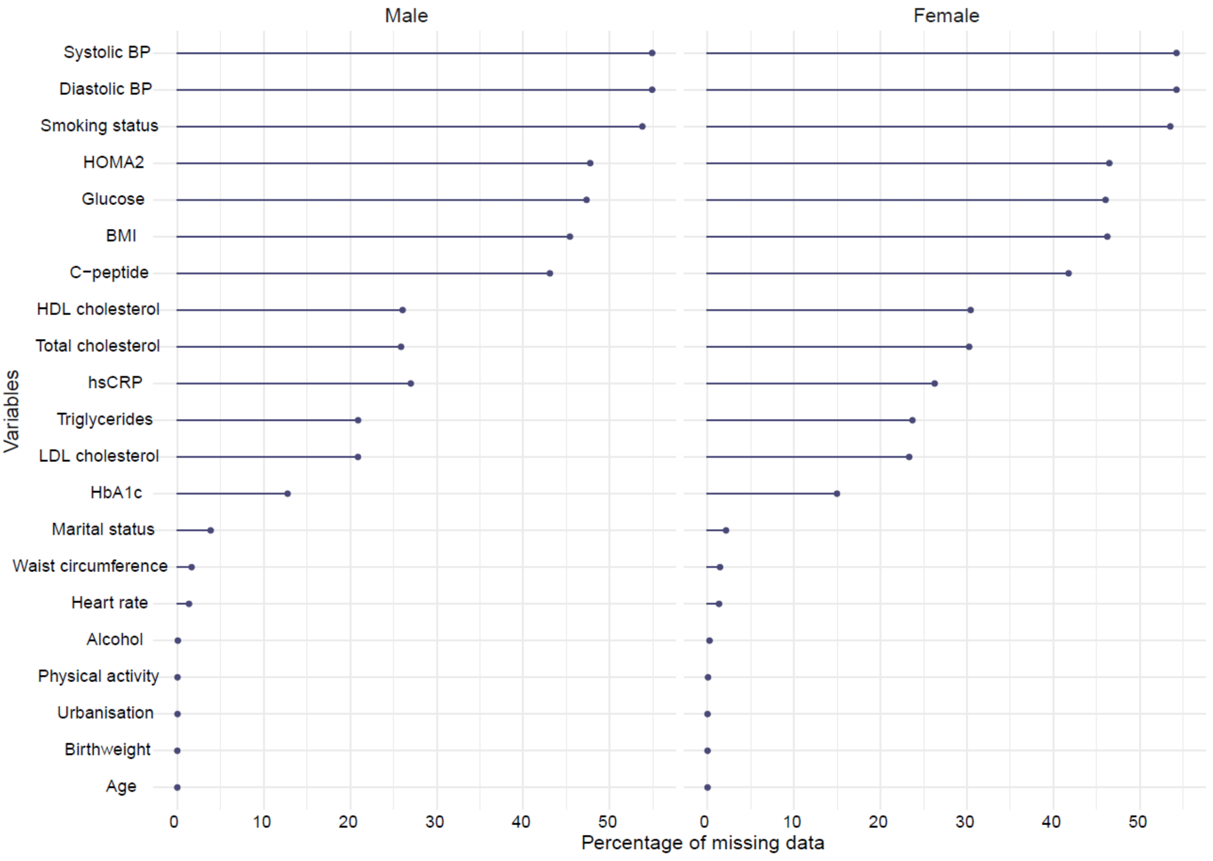
Male
Female
Systolic BP
Diastolic BP
Smoking status
HOMA2
Glucose
BMI
C−peptide
HDL cholesterol
Total cholesterol
hsCRP
Triglycerides
LDL cholesterol
HbA1c
Marital status
Waist circumference
Heart rate
Alcohol
Physical activity
Urbanisation
Birthweight
Age
Variables
0 10 20 30 40 50
Percentage of missing data

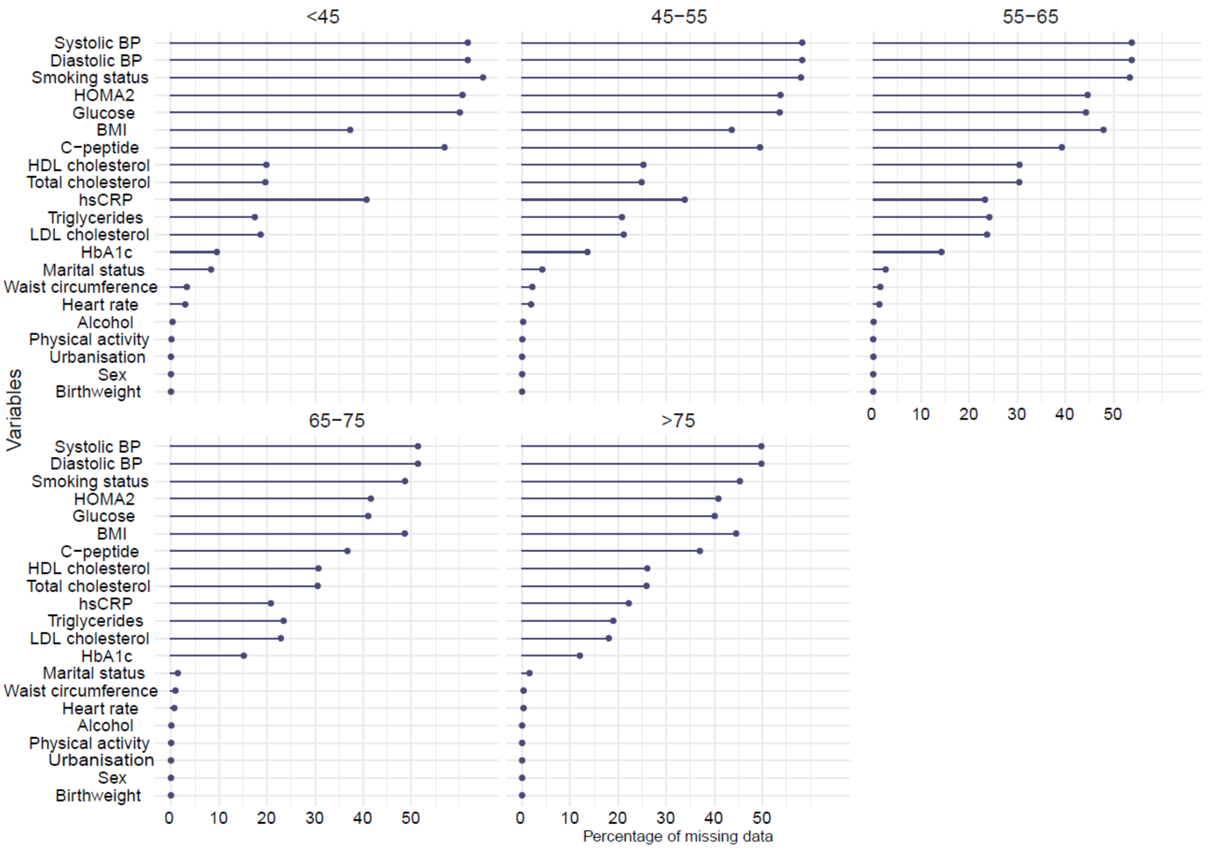
<45
45−55
55−65
65−75
>75
Systolic BP
Diastolic BP
Smoking status
HOMA2
Glucose
BMI
C−peptide
HDL cholesterol
Total cholesterol
hsCRP
Triglycerides
LDL cholesterol
HbA1c
Marital status
Waist circumference
Heart rate
Alcohol
Physical activity
Urbanisation
Sex
Birthweight
Variables
0 10 20 30 40 50
Percentage of missing data

Legend: Percentage of missing data of variables used in the study. Each birthweight group is in kilograms. Abbreviations: BMI = Body Mass Index, HDL = high-density lipoprotein, LDL = low-density lipoprotein, hsCRP = high-sensitivity C-reactive protein, HOMA2 = Homeostasis Model Assessment 2, T2D = type 2 diabetes. Comorbidities and medication do not have any missing data.

We used multiple imputation to create and analyse 20 imputed datasets, with the number of iterations set to 10. Incomplete variables were imputed under fully conditional specification, using the default settings of the mice 3.14.0 package[2]. The parameters of substantive interest were estimated in each imputed dataset separately and combined using Rubin's rules.

The following variables were used in the imputation model: age at enrolment, sex, alcohol consumption, physical activity, marital status, urbanization (level of urbanization), weight, waist, height, BMI, family history of type 2 diabetes, HOMA2-Beta, HOMA2 insulin sensitivity, C-peptide, blood glucose, hsCRP, birthweight, born-at-term status, total cholesterol, LDL cholesterol, HDL cholesterol, triglycerides, smoking status, systolic blood pressure, diastolic blood pressure, HbA_{1c}, number of glucose-lowering drugs, type of glucose-lowering drugs, lipid-lowering drugs, antihypertensive medication, number of antihypertensive drugs, calendar year at birth, and the outcomes of interest.

The following variables were imputed by the following methods:

For continuous variables, predictive mean matching was used and included weight, waist, height, BMI, HOMA2-Beta, HOMA2 insulin sensitivity, C-peptide, blood glucose, hsCRP, total cholesterol, HDL cholesterol, LDL cholesterol, triglycerides, systolic blood pressure, diastolic blood pressure, and HbA_{1c}. For binary data logistic regression, imputation was used and included born-at-term status. For categorical data with more than two levels, polytomous regression imputation was used and included smoking status and marital status.

We combined the raw data variables of weight and height measurements into BMI before the imputation procedure. This procedure was justified by our findings that inclusion of only the raw variables for weight and height, gave rise to imprecise imputations, as outlines in a previous study[3]. Overall, the observed and imputed values had similar distributions, as shown in the density plots below.

Density plot of imputations:

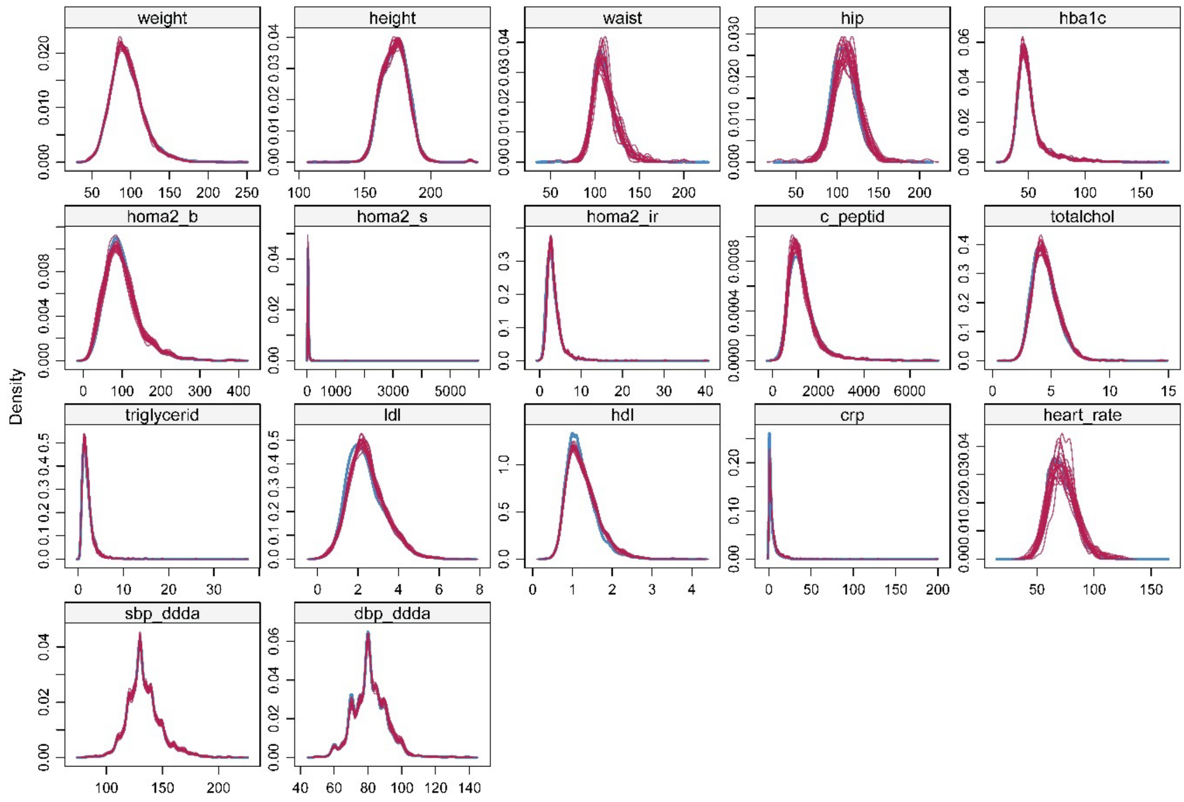

Red = Imputations, Blue = Original data.

Variables in plot: weight; waist = waist circumference; height; hip = hip circumference; homa2_b = HOMA2 Beta; homa2_s = HOMA2-insulin sensitivity; homa2_ir = HOMA2-insulin resistance; c_peptid = C-peptide; crp= C-reactive protein; totalchol = total cholesterol; hdl = HDL cholesterol; sbp_ddda = systolic blood pressure; dbp_ddda = diastolic blood pressure; hba1c_nip = HbA_{1c}; ldl = LDL cholesterol; triglycerid = triglycerides; heart_rate = resting heart rate.

Exploring birthweight as a continuous exposure using restricted cubic spline regression.
Spline knots were placed at fixed quantiles of the predictor's marginal distribution[13, 14]. Best models were chosen by visual inspection and lowest Akaike Information Criterion (AIC). All models were adjusted for sex, calendar year of birth, age at enrolment, family history of type 2 diabetes, and born-at-term status.

Default quantiles for knots placement of restricted cubic spline models:

k	Birthweights (g)
3	2750, 3400, 4000
4	2500, 3200, 3550, 4250
6	2500, 3000, 3250, 3500, 3750, 4250
8	2200, 2900, 3100, 3300, 3500, 3625, 3900, 4450

Composite CVD endpoint

	**AIC**
Linear	21469
2-degree polynomial	21470
3-degree polynomial	21472
Restricted cubic spline: 3 knots	21470
Restricted cubic spline: 4 knots	21588
Restricted cubic spline: 6 knots	21590
Restricted cubic spline: 8 knots	21591

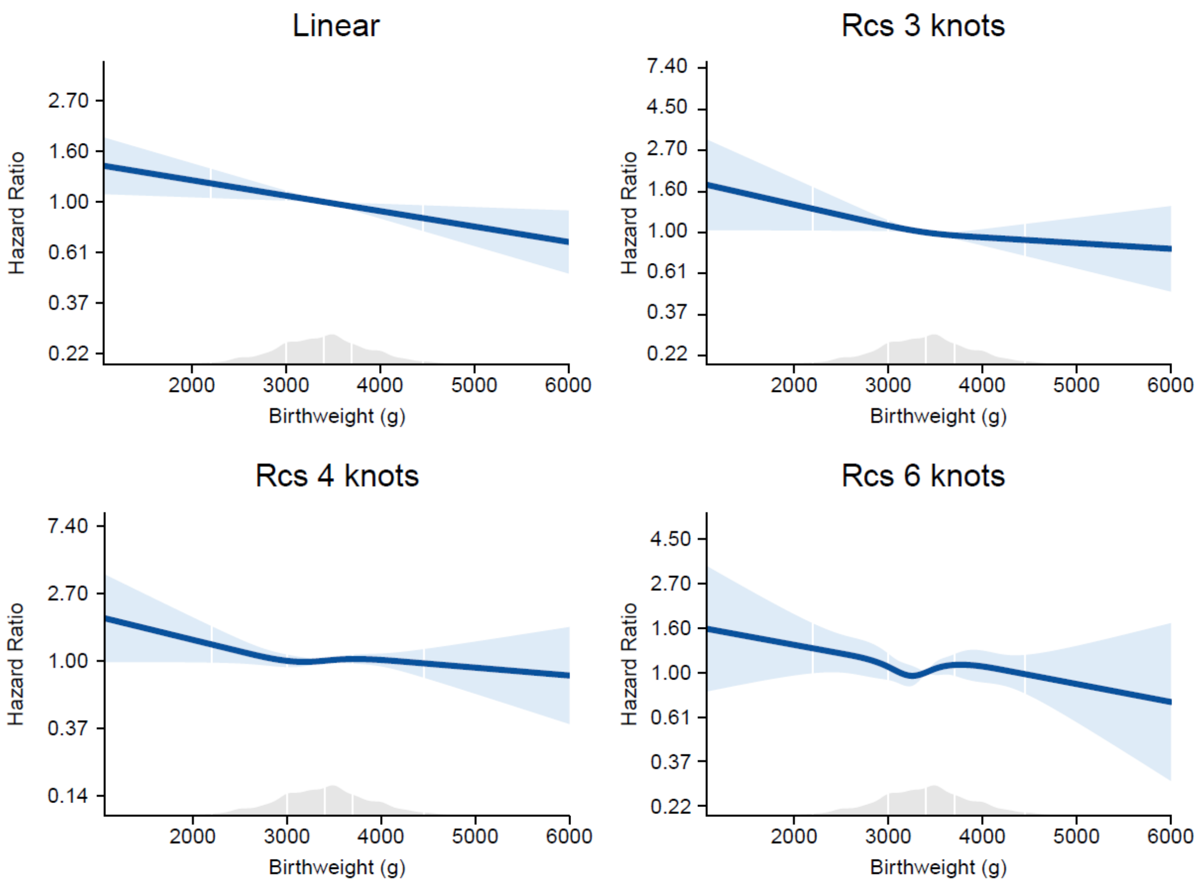

MACE

MACE	**AIC**
Linear	14546
2-degree polynomial	14547.75
3-degree polynomial	14549.46
Restricted cubic spline: 3 knots	14547.01
Restricted cubic spline: 4 knots	14548
Restricted cubic spline: 6 knots	14546
Restricted cubic spline: 8 knots	14549.19

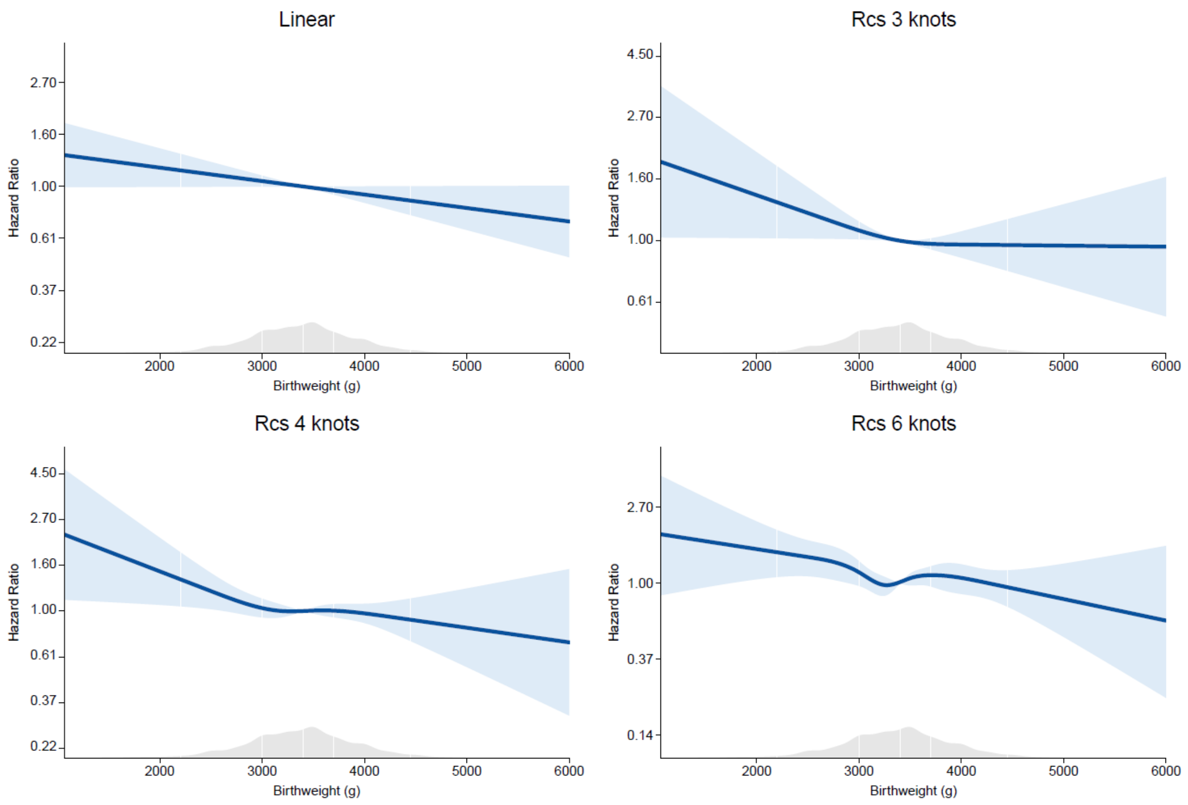

All-cause mortality

All-cause mortality	**AIC**
Linear	17500
2-degree polynomial	17502.75
3-degree polynomial	17504.59
Restricted cubic spline: 3 knots	17502.8
Restricted cubic spline: 4 knots	17502.36
Restricted cubic spline: 6 knots	17504.92
Restricted cubic spline: 8 knots	17504.23

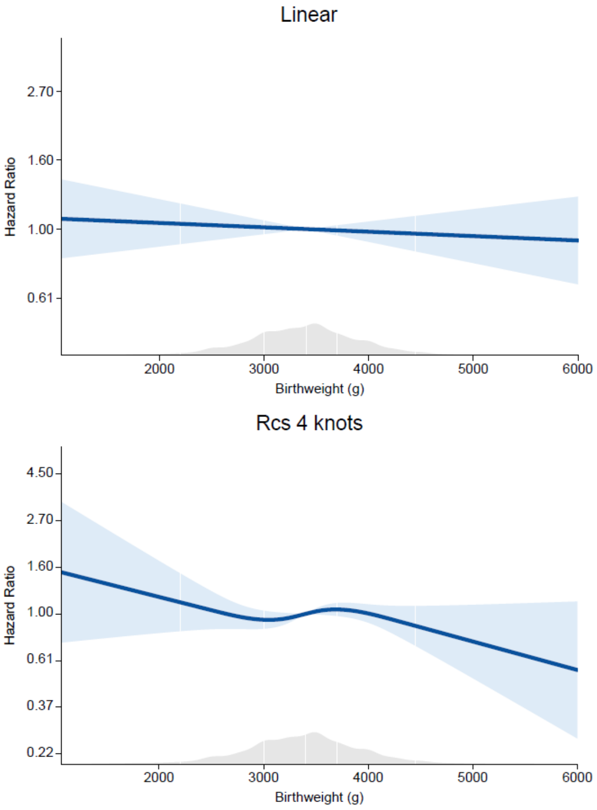
Linear
Rcs 3 knots
Rcs 4 knots
Rcs 6 knots
Hazard Ratio
Birthweight (g)
2000
3000
4000
5000
6000
4.50
2.70
1.60
1.00
0.61
0.37
0.22

Exploring sex stratified analysis with birthweight as a continuous exposure using restricted cubic spline regression.

Spline knots were placed at fixed quantiles of the predictor's marginal distribution[13, 14]. Best models were chosen by visual inspection and lowest Akaike Information Criterion (AIC). All models were adjusted for sex, calendar year of birth, age at enrolment, family history of type 2 diabetes, and born-at-term status.

Default quantiles for knots placement of restricted cubic spline models:

k	Birthweights (g)	
	Female	Male
3	2750, 3400, 4000	2790, 3450, 4100
4	2500, 3200, 3550, 4250	2500,3250, 3600, 4300

Composite CVD endpoint

	**AIC**	
	**Female**	**Male**
Linear	5568	14369
Restricted cubic spline: 3 knots	5569	14371
Restricted cubic spline: 4 knots	5570	14373

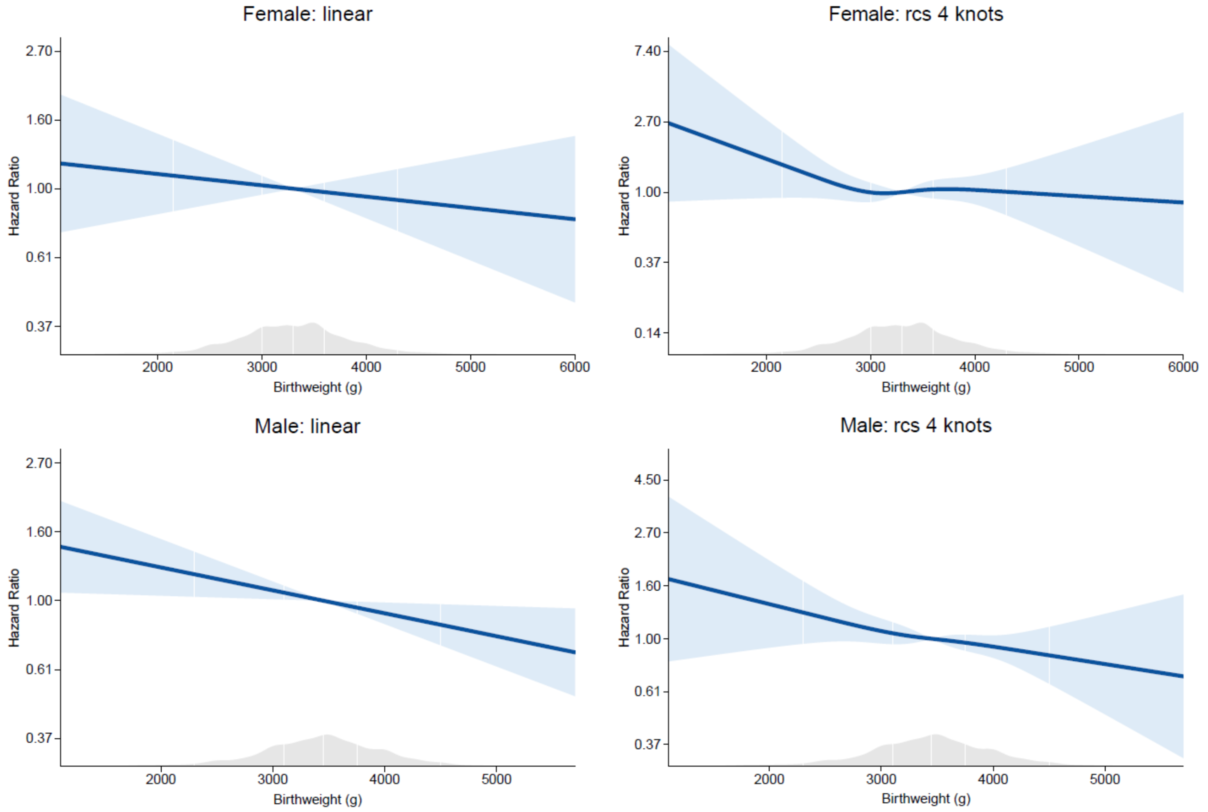

MACE

	AIC	
	**Female**	**Male**
Linear	4365	11847
Restricted cubic spline: 3 knots	4363	11849
Restricted cubic spline: 4 knots	4361	11851

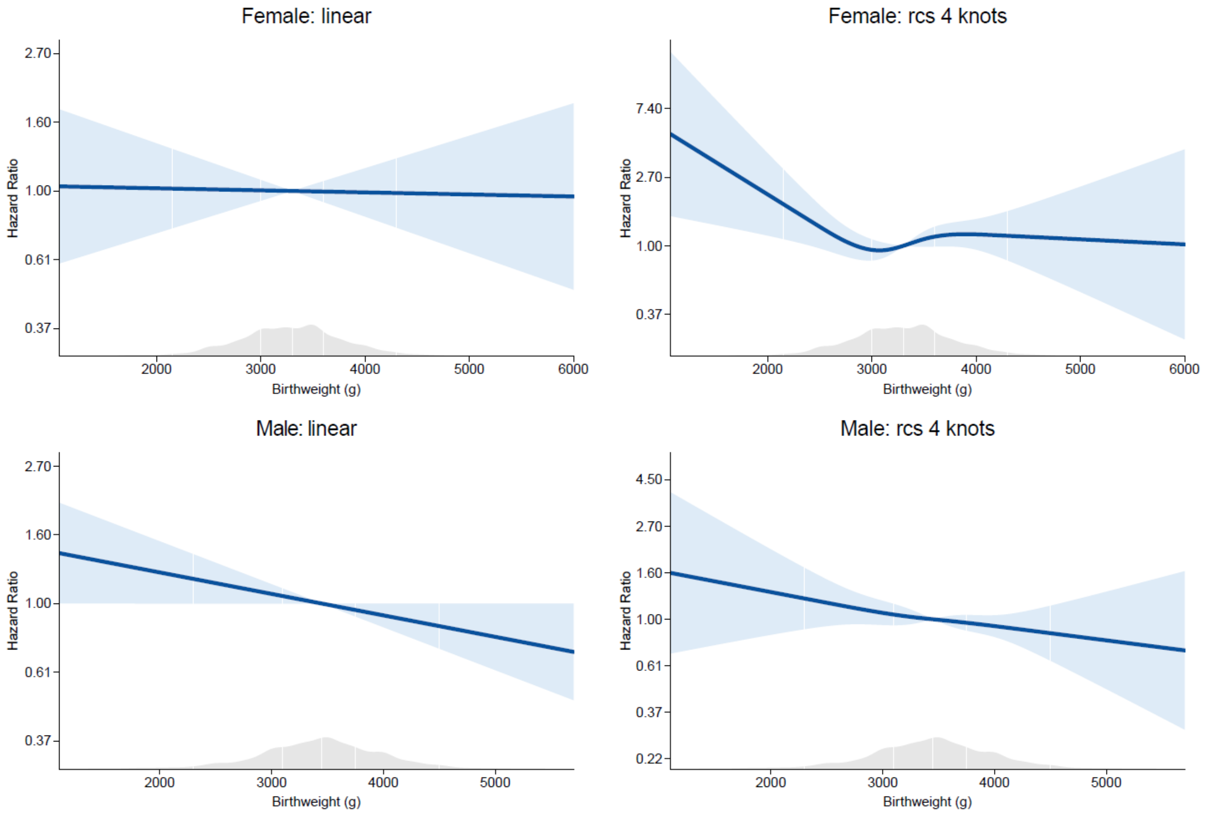

All-cause mortality

	AIC	
	**Female**	**Male**
Linear	5762	12353
Restricted cubic spline: 3 knots	5764	12354
Restricted cubic spline: 4 knots	5766	12352

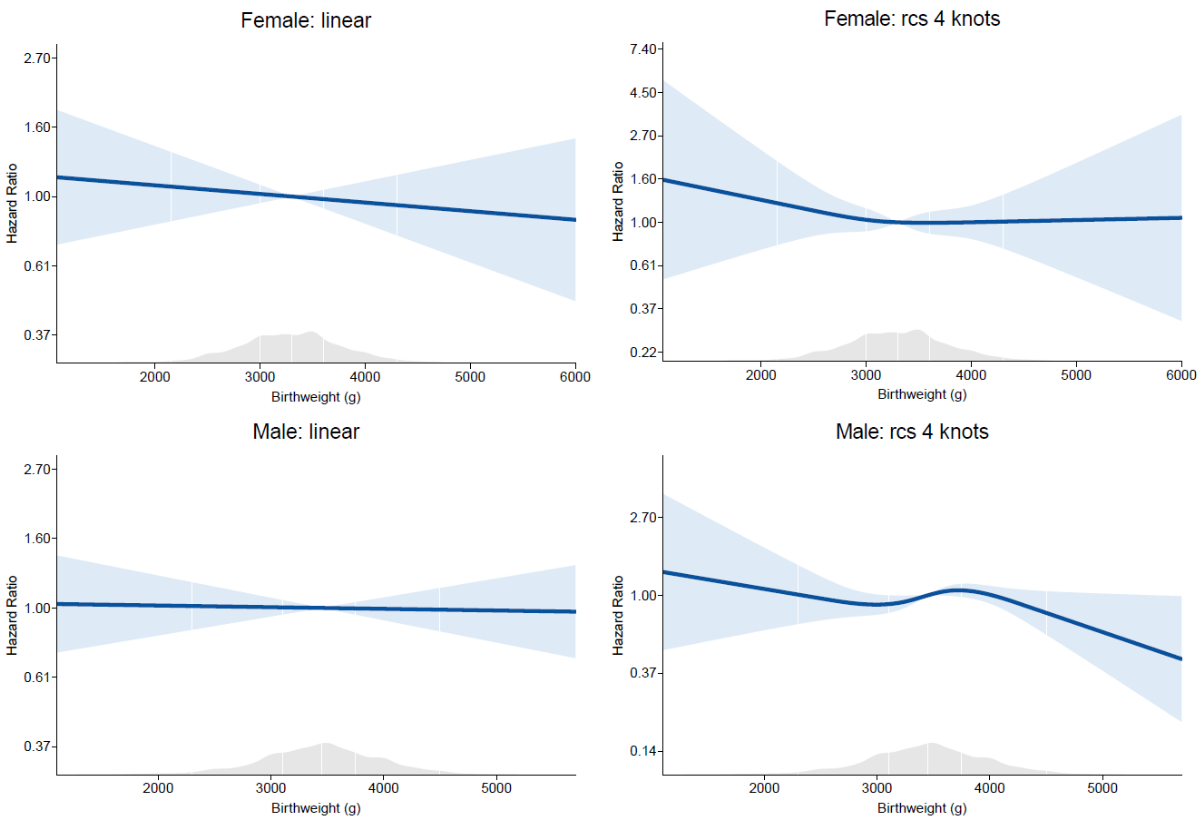
Female: linear
Female: rcs 4 knots
Male: linear
Male: rcs 4 knots
Hazard Ratio
Birthweight (g)
2.70
1.60
1.00
0.61
0.37
7.40
4.50
0.22
0.14
2000
3000
4000
5000
6000

ESM Tables

ESM table 1: data sources

Data sources	Description
DD2 biobank[4]	DD2 is a prospective, nationwide population-based cohort of recently diagnosed type 2 diabetes individuals, with collection of matching interview data and biological samples for a biobank at baseline. Enrolment started in November 2010 and is still ongoing.
DD2 questionnaire[5]	
The Danish Adult Diabetes Registry[6] (DDDA)	The database was established to assess quality in diabetes care on a national level in 2004. It only covers a subset of the diabetes population.
The Danish National Patient Registry[7]	Covers all inpatient (somatic) hospital contacts since 1977 and from 1995 all inpatient and outpatient hospital contacts in Denmark. Diagnostic information is coded according to the International Classification of Diseases, Tenth Revision (ICD-10) from 1994 onwards. We retrieved information from 1994 and onwards.
The Danish National Prescription Registry[8] (DNHSP)	Covers all redeemed prescriptions at Danish pharmacies since 1995.
Civil Registration System[9]	All citizens of Denmark are registered in this system by a unique Civil Personal Register number linked to registrations of birth, address, marital status, kinship, and migration, among others. The Civil Personal Register number is used as linkage to other databases.
The Danish Registry of Causes of Death[10]	Death and cause of death among citizens in Denmark are recorded in this database since 1970. Both the immediately cause and the underlying cause of death are registered. Causes of death were coded according to the International Classification of Diseases, Tenth Revision (ICD-10) from 1994 onwards.
The Danish Medical Birth Register[11]	The register includes data on all birth in Denmark since 1973. It contains information of the health of pregnant women and their offspring.
National Lab Database (Laboratoriedatabasen)	The National Lab Database is comprised of detailed information of all laboratory tests carried out at Denmark’s larger biochemical and immunological laboratories. It started in 2008 and complete coverage of the different regions in Denmark’s starting from 2010.

ESM Table 2: Variable definitions

Variable definitions	Source	Remarks
Biological sex	Civil Registration System	Categorical variable: male or female
Age at enrolment	Civil Registration System and DD2	Age at study enrolment in DD2<br>Continuous variable (years)<br>Categorical variable:<br><45<br>45-55<br>55-65<br>65-75<br>>75
Age at diagnosis	Civil Registration System and DD2	Defined as age as whichever of the following events came first:<br>1. First prescription for glucose-lowering drugs<br>2. First diabetes-related diagnosis in the Danish National Patient Registry<br>3. DDDA registration<br>4. First measured $HbA1_c \geq 48$ mmol/mol<br>5. DD2 enrolment<br>Continuous variable (years)
Family history of type 2 diabetes	DD2 questionnaire	Self-reported at DD2 study enrolment. Comprised of mother, father, grandparents, and sibling<br>Categorical variable (number of affected relatives)
Enrolment status	DD2	Patients were enrolled either at general practitioners or hospital outpatient clinics. Categorical variable
Born-at-term status	Danish National Archive	Midwifes' answers to the question "born at term" in the Midwife Record. The answers may be associated with some uncertainty, as the accuracy of information depends on the midwife's clinical judgement and because information regarding whether an individual was "born at term" could be written in different ways, with some changes over time. The midwife records usually had one check box for "born at term" and another one for "preterm". If the midwife erroneously wrote "yes" in both boxes, this counted as missing data. For children registered as "preterm", midwives could further assess and write in the record how long before term the child was born. This information was often either missing or given as a range (e.g., "from 1 to 4 weeks early"), and we therefore chose not to use the data to evaluate the exact gestational age. Instead, we constructed an indicator variable "born-at-term: yes/no", from the check box of "born at term".
Weight at enrolment	DD2 or DDDA	If weight recorded as part of the DD2 enrolment process is available (few [not part of the DD2 core data initially]), we used that weight measure, otherwise the DDDA weight closest to DD2 enrolment,

		with a maximum of 2 years prior to enrolment.<br>Weights below 35 and above 300 kg were considered outliers.<br>Continuous variable (kg)
Height	DD2, DD2 2016 questionnaire or DDDA	Data on height is available from three different sources:<br>DD2 enrolment, DDDA data (repeated measures), DD2 Diabetic neuropathy questionnaire 2016 (self-reported).<br><br>Regarding DDDA data: a mean height based on all available DDDA heights were calculated for all patients 18 years or older.<br><br>We do not expect height to change over time among these adults.<br>Thus, we used the available heights in hierarchically order; height from DD2 enrolment (measured by health personal, but very few available), DD2 questionnaire survey in 2016, DDDA.<br>Heights below 130 cm and above 220 were considered outliers and not included in any calculations.<br>Continuous variable (cm)
Body mass index (BMI) at enrolment	DD2, DD2 2016 questionnaire and/or DDDA	BMI were calculated using standard formula of weight/height (in meter)^2<br>BMI below 15kg/m^2 and above 70kg/m^2 were considered outliers and not included in any calculations.<br>Continuous variable (kg/m^2)
Waist circumference	DD2	Measured at DD2 enrolment.<br>Waist circumference below 50cm and above 184cm were considered outliers and not included in any calculations.<br>Continuous variable (cm)
Hip circumference	DD2	Measured at DD2 enrolment.<br>Hip circumference below 44cm and above 175cm were considered outliers and not included in any calculations.<br>Continuous variable (cm)
Alcohol consumption	DD2	Self-reported at enrolment. Units/week<br><br>High risk intake: >14/21 drinks per week for female/male.<br>High-risk alcohol consumption was categorized according to the Danish Health Authority's definitions as more than 21 and 14 drinks weekly for men and women, respectively in 2010 when the DD2 was initiated.<br>Categorical variable
Smoking status	DDDA	Self-reported. Collected from DDDA, using the value closest to DD2 enrolment date and a maximum 2 years prior to enrolment.<br>Categories:<br>• Never smoker<br>• Former smoker

		• Current smoker (comprised of daily and occasionally)
Physical activity	DD2	Self-reported at DD2 enrolment: "days per week of minimum 30 minutes of moderate to vigorous activity"<br>Categorical variable (days per week)
Marital status	Civil Registration System	Collected from the Civil Registration System, using the most recent registration prior to the DD2 enrolment date.<br>Categories:<br>• Married/registered partnership<br>• Divorced/separated<br>• Widow/widower<br>• Never married/registered partnership
Level of urbanization	Statistics Denmark	Collected from Statistics Denmark, using the most recent registration prior to the DD2 enrolment date. Urbanization levels were categorized according to municipality level groups defined by Statistics Denmark - Municipality Groups v1:2018- - Statistics Denmark (dst.dk)<br>Five levels exist and are based on 1: the size of the largest city in the municipality and 2: availability of jobs (direct and indirect). Municipalities with ≥ 200,000 jobs are capital municipalities. Municipalities with <200,000 jobs and ≥100,000 residents are defined as large city municipalities. Municipalities with <200,000 jobs and between 30,000-100,000 residents are provincial municipalities. Municipalities with 40,000-200,000 jobs and <30,000 residents are Surrounding area municipalities. The remaining municipalities are defined as rural municipalities.
Systolic blood pressure	DDDA	Collected from DDDA, using the value closest to DD2 enrolment date and maximum 2 year prior to enrolment. Systolic blood pressure below 90mmHg and above 260mmHg were considered outliers and not included in any calculations.<br>Continuous variable (mmHg)
Diastolic blood pressure	DDDA	Collected from DDDA, using the value closest to DD2 enrolment date and maximum 2 year prior to enrolment. Diastolic blood pressure below 30mmHg and above 140mmHg were considered outliers and not included in any calculations.<br>Continuous variable (mmHg)
Total cholesterol	DDDA,<br>National Lab database (NPU-codes: NPU01566, NPU18412)	Collected from DDDA and the National Lab database, using the value closet to enrolment date and maximum 2 year prior to enrolment.<br>Total cholesterol above 9.87mmol/L were considered outliers and not included in any

		calculations.<br>Continuous variable (mmol/L)
Triglycerides	DDDA,<br>National Lab database (NPU-codes: NPU04094, NPU03620)	Collected from DDDA and the National Lab database, using the value closet to enrolment date and maximum 2 year prior to enrolment.<br>Triglycerides above 9.30 mmol/L were considered outliers and not included in any calculations.<br>Continuous variable (mmol/L)
HDL cholesterol	DDDA,<br>National Lab database (NPU-codes: NPU01567, NPU10157)	Collected from DDDA and the National Lab database, using the value closet to enrolment date and maximum 2 year prior to enrolment.<br>HDL cholesterol above 3.08 mmol/L were considered outliers and not included in any calculations.<br>Continuous variable (mmol/L)
LDL cholesterol	DDDA,<br>National Lab database (NPU-codes: NPU01568, NPU10171, DNK35308)	Collected from DDDA and the National Lab database, using the value closet to enrolment date and maximum 2 year prior to enrolment.<br>LDL cholesterol above 7 mmol/L were considered outliers and not included in any calculations.
Blood glucose	DD2 biobank	Measured at DD2 enrolment.<br>Continuous variable (mmol/L)
HbA1c	DDDA, National Lab database (NPU-codes: NPU03835, NPU27300)	Collected from DDDA and the National Lab database, using the value closet to enrolment date and maximum 2 year prior to enrolment.<br>HbA1c above 15% were considered outliers and not included in any calculations.<br>Continuous variable (mmol/mol (%))
C-peptide	DD2 biobank	Measured at DD2 enrolment.<br>Continuous variable (pmol/L).<br>C-peptide above 4672 pmol/L were considered outliers and not included in any calculations.
hsCRP	DD2 biobank	High sensitivity low-grade inflammation.<br>Measured at DD2 enrolment.
GAD-Antibody	DD2 biobank	Measured at DD2 enrolment.
HOMA2<br>- Insulin sensitivity<br>- Beta	DD2 biobank	We used the revised homeostatic assessment model version 2 (HOMA2) to estimate insulin sensitivity (HOMA2S) and insulin secretion (HOMA2B) based on fasting C-peptide and plasma glucose values, based on the DD2 biobank[12].<br>HOMA2-sensitivity above 512 and HOMA2-Beta above 320 were considered outliers and not included in any calculations.
Antihypertensive drug usage	DNHSP	Up to one year prior to enrolment. Classes and ATC codes:<br>**ACE inhibitors or angiotensin II receptor antagonists:** C09A, C09B, C09C, C09D, C10BX04, C10BX06, C10BX07, C10BX11, C10BX12, C10BX13, C10BX14, C10BX15, C10BX10

		**Calcium channel antagonists:** C08, C09BB. C09DB, C09DX01, C09DX03, C09XA53, C09XA54, C07FB, C09BX01, C09BX03, C10BX07, C10BX09, C10BX11, C10BX14<br>**Low-ceiling diuretics:** C03A, C03B, C03EA, C07D, C09BA, C09DA, C09XA52, C09XA54, C08G, C07B, C09DX01, C09DX03, C09BX03<br>**Potassium-sparing diuretics:** C03D, C03E<br>**Beta-blockers:** C07<br>**Alpha-blockers:** C02CA04, C04CA03<br>**Central adrenergic inhibition:** C02AC05, C02AB<br>**Renin-inhibitors:** C09XA, C09DX02
Number of antihypertensive medications	DNHSP	Up to one year prior to enrolment.<br>One or more hits in one group counts as one agent: Thiazides, potassium-sparing diuretics, beta-blockers, Calcium channel antagonists, ACE inhibitors or ATII antagonists, Renin inhibitors, Alpha-blockers (Doxazosin), Central adrenergic inhibition (Monoxidin, methyldopa).
Lipid lowering drug usage	DNHSP	Up to one year prior to enrolment.<br>ATC: C10, A10BH51
GLP-1-analouge or SGLT2 inhibitor usage	DNSHP	GLP1-analogue or SGLT2 inhibitor use Yes/No<br>ATC:<br>**GLP-1 analogues:** A10BX04, A10BX07, A10BX10, A10BX13, A10BX14, A10BJ, A10AE54, A10AE56<br>**SGLT2-inhibitors:** A10BX09, A10BX11, A10BX12, A10BD15, A10BD16, ADBD19, A10BD20, ADBD21, A10BK, A10BD23, A10BD24
Insulin usage	DNHSP	Insulin use yes/no. Up to one-year prior enrolment.<br>ATC: A10A
Other glucose-lowering medication	DNSHP	other glucose-lowering (metformin, **DPP-4 inhibitors, SU and Meglitinides, Thiazolidinediones and Alfa-glucosidase inhibitors)** yes/No (A10)<br>Classes and ATC codes:<br>**Metformin:** A10BA, A10BD02, A10BD03, A10BD05, A10BD07, A10BD08, A10BD10, A10BD11, A10BD13, A10BD14, A10BD15, A10BD16, A10BD17, A10BD18, A10BD20, A10BD22<br>**DPP-4 inhibitors:** A10BH, A10BD07, A10BD08, A10BD09, A10BD10, A10BD11, A10BD12, A10BD13, A10BD18, A10BD19, A10BD21, A10BD22<br>**SU and meglitinides:** A10BB, A10BD04, A10BD02, A10BD06, A10BD01, A10BC01. A10BX02, A10BX03, A10BX08, A10BD14

		**Thiazolidinediones:** A10BG, A10BD03, A10BD04, A10BD05, A10BD06, A10BD09, A10BD12<br>**Alfa-glucosidase inhibitors:** A10BF, A10BD17
Number of different antidiabetics including insulin	DNHSP	Up to one year prior to enrolment.<br>One hit or more hits in one group counts as one agent: Sulfonylureas, Meglitinides, Metformin, alpha-glucosidase inhibitors, Glitazones, DD4-inhibitors, GLP-1 analogues, SGLT-2 inhibitors, Fast-acting insulin, Long-acting insulin
Any cardiovascular complication prior to index date	The Danish National Patient Registry	Any registration of primary or secondary inpatient diagnoses or operation codes (regardless of hospitalization) prior to index date, with a look-back period from DD2 enrolment to 1994, of the following:<br>DI21, DI23, DI24, DT822A (ischemic heart disease); DT823 (acute ischemic heart disease with/without complications); DI20 (angina pectoris); DI25 (chronic ischemic heart disease); KFNA, KFNB, KFNC, KFND, KFNE, KFNF, KFNG, KFNH, KFNW, KFLF (coronary bypass or percutaneous coronary intervention); DI500, DI501, DI502, DI503, DI508, DI509, DI110, DI130, DI132, DI420, DI426, DI427, DI428, DI429 (heart failure); DI48 (atrial fibrillation/flutter); DI61 (cerebral bleeding); DI63, DI64, DI65, DI66 (cerebrovascular infarct); DG45 (transient cerebrovascular disease); DI672, DI678, DI679 (unspecified cerebrovascular disease); DI691, DI693, DI694, DI698 (previous cerebrovascular disease); KAAL10, KAAL11 (cerebral thrombolysis or thromboendarterectomy)<br>DE105, DE115, DE125, DE135, DE145 (diabetes with peripheral vascular complications); DI700, DI701, DI702, DI708, DI709, DI739, DI74, DN280, DK550, DK551, DH340, DH341, DH342 (peripheral/abdominal vascular disease); KNBQ, KNCQ, KNDQ, KNEQ, KNFQ, KNGQ, KNHQ, KPAE, KPAF, KPAH, KPAN, KPAP, KPAQ, KPAW99, KPAU74, KPBE, KPBF, KPBH, KPBN, KPBP, KPBQ, KPBW, KPGH10, KPCE, KPCF, KPCH, KPCN, KPCP, KPCQ, KPCW99, KPCW20, KPCU74, KPCU82, KPCU83, KPCU84, KPGE, KPGF, KPGH, KPGN, KPGP, KPGQ, KPGW99, KPGW20, KPEE, KPEF, KPEH, KPEN, KPEP, KPEQ, KPEW, KPFE, KPFH, KPFN, KPFP, KPFQ, KPFW, KPDU74, KPDU82, KPDU83, KPDU84, KPEU74, KPEU82, KPEU83, KPEU84, KPFU74, KPFU82, KPFU83, KPFU84, KPGU74, KPGU83, KPGU84, KPGU99 (vascular surgery)

P-HbA1c	National Lab database	NPU/DNK codes:<br>NPU03835: in % (9626)<br>NPU27300: mmol/mol (235,110)<br><br>Transformation to mmol/mol has been made by the following formula:<br>HbA1c (%) * 10.93 – 23.5
P-C-reactive protein	National Lab database	NPU/DNK codes:<br>NPU19748: mg/l (162,122)
P-Triglycerides	National Lab database	NPU/DNK codes:<br>NPU04094: mmol/l (128,078)<br>NPU03620: mmol/l (27,075)
P-Total cholesterol	National Lab database	NPU/DNK codes:<br>NPU01566: mmol/l (153,539)<br>NPU18412: mmol/l (2112)
P-HDL	National Lab database	NPU/DNK codes:<br>NPU01567: mmol/l (151,645)<br>NPU10157: mmol/l (2105)
P-LDL	National Lab database	NPU/DNK codes:<br>NPU01568: mmol/l (112,728)<br>NPU10171: mmol/l (9917)<br>DNK35308: mmol/l (24,899)

ESM Table 3: Outcome definitions

Variables	Definition	Source
All-cause mortality	Death from any cause	The Civil Registration System
Composite cardiovascular endpoint (CVD)	First occurrence of myocardial infarction, stroke, unstable angina, coronary revascularization, heart failure hospitalization, peripheral revascularization, or cardiovascular death. See definitions below	
3-point Major Adverse Cardiovascular Event (MACE)	First occurrence of myocardial infarction, stroke, cardiovascular death, and coronary revascularization. See definitions below.	
Myocardial infarction	DI21<br>and<br>Coronary revascularization: KFNA, KFNB, KFNC, KFND, KFNE, KFNF, KFNG, KFNH20	Primary or secondary inpatient discharge ICD-10 diagnoses from the Danish National Patient Registry (date defined by d_inddto) and ICD-10 procedure codes (regardless of hospitalization) from the Danish National Patient Registry (date defined by d_odto)
Unstable angina pectoris	DI200	Primary or secondary inpatient discharge ICD-10 diagnoses from the Danish National Patient Registry (date defined by d_inddto)
Stroke	DI61, DI63, DI64	Primary or secondary inpatient discharge ICD-10 diagnoses from the Danish National Patient Registry (date defined by d_inddto)
Heart failure	DI50, DI110, DI130, DI132	Primary or secondary inpatient, emergency room or outpatient discharge ICD-10 diagnoses from the Danish National Patient Registry (date defined by d_inddto) and no prior diagnosis of heart failure
Atrial fibrillation	DI48	Primary or secondary inpatient, emergency room or outpatient discharge ICD-10 diagnoses from the Danish National Patient Registry (date defined by d_inddto) and no prior diagnosis of atrial fibrillation/flutter
Peripheral revascularization (lower limb including intrarenal aorta and a. iliacae)	DI739, DI702<br>and<br>Thrombectomy or embolectomy: KPDE, KPEE, KPFE<br>Thromboendarterectomy: KPDF, KPEF, KPFF, KPDU74. KPEU74, KPFU74<br>bypass-operations: KPDH, KPEH, KPFH, KPGH20+21+22+23+30+31+40+99<br>Angioplasty: KPDN, KPEN, KPFN KPDU82, KPEU82, KPFU82	Primary or secondary inpatient discharge ICD-10 diagnoses from the Danish National Patient Registry (date defined by d_inddto) and ICD-10 procedure codes (regardless of hospitalization) from the Danish National Patient Registry (date defined by d_inddto and d_odto)

	Percutaneous angioplasty: KPDP, KPEP, KPFP, KPDU83, KPEU83, KPFU83<br>Unspecified: KPDW, KPEW, KPFW	
Cardiovascular death	DI00-DI99 from Danish Registry of Causes of Death OR death from The Civil Registration System AND within 30 days a diagnosis of:<br>Myocardial infarction: DI21,<br>Unstable Angina: DI200,<br>Stroke: DI61, DI63, DI64,<br>Heart failure Hospitalization: DI50, DI110, DI130, DI132,<br>Coronary revascularization: KFNA, KFNB, KFNC, KFND, KFNE, KFNF, KFNG, KFNH20,<br>Peripheral revascularization:<br>Thrombectomy or embolectomy: KPDE, KPEE, KPFE<br>Thromboendarterectomy: KPDF, KPEF, KPFF, KPDU74. KPEU74, KPFU74<br>bypass-operations: KPDH, KPEH, KPFH, KPGH20+21+22+23+30+31+40+99<br>Angioplasty: KPDN, KPEN, KPFN KPDU82, KPEU82, KPFU82<br>Percutaneous angioplasty: KPDP, KPEP, KPFP, KPDU83, KPEU83, KPFU83<br>Unspecified: KPDW, KPEW, KPFW,<br>Complications after myocardial infarction (DI24)<br>Chronic ischemic heart disease (DI25)<br>Cardiac arrest/sudden cardiac death (DI46)<br>Ventricular tachycardia (DI472)<br>Ventricular fibrillation/flutter (DI490)	Death was obtained from the Civil Registration System. The cause of death (either immediate or underlying causes) was obtained from the Danish Registry of Causes of Death. Cardiovascular events were obtained by primary or secondary inpatient or outpatient discharge ICD-10 diagnoses from the Danish National Patient Registry (date defined by 30 days from d_inddto)

ESM Table 4: Medication according to birthweight

Variable	**<3000 g (n=2,152)**	**3000–3700 g (n=4,262)**	**>3700 g (n=2,003)**	**Total (n=8,417)**
Antihypertensive medication				
No	627 (29.1)	1,205 (28.3)	548 (27.4)	2,380 (28.3)
Yes	1,525 (70.9)	3,057 (71.7)	1,455 (72.6)	6,037 (71.7)
Antihypertensive medication excl. loop diuretics				
No	653 (30.3)	1,262 (29.6)	564 (28.2)	2,479 (29.5)
Yes	1,499 (69.7)	3,000 (70.4)	1,439 (71.8)	5,938 (70.6)
ACE-inhibitors/AT2				
No	1,098 (51.0)	2,126 (49.9)	1,008 (50.3)	4,232 (50.3)
Yes	1,054 (49.0)	2,136 (50.1)	995 (49.7)	4,185 (49.7)
Number of antihypertensive medication (n)				
0	653 (30.3)	1,262 (29.6)	564 (28.2)	2,479 (29.5)
1	604 (28.1)	1,083 (25.4)	539 (26.9)	2,226 (26.5)
2	480 (22.3)	1,071 (25.1)	487 (24.3)	2,038 (24.2)
3+	415 (19.3)	846 (19.9)	413 (20.6)	1,674 (19.9)
Lipid-lowering medication				
No	668 (31.0)	1,305 (30.6)	623 (31.1)	2,596 (30.8)
Yes	1,484 (69.0)	2,957 (69.4)	1,380 (68.9)	5,821 (69.2)
Glucose-lowering medication				
No use	267 (12.4)	584 (13.7)	274 (13.7)	1,125 (13.4)
oral only	1,732 (80.5)	3,416 (80.2)	1,604 (80.1)	6,752 (80.2)
insulin+oral/insulin only	129 (6.0)	226 (5.3)	109 (5.4)	464 (5.5)
insulin only	24 (1.1)	36 (0.8)	16 (0.8)	76 (0.9)
GLP1/SGLT2				
No	1,920 (89.2)	3,879 (91.0)	1,852 (92.5)	7,651 (90.9)
Yes	232 (10.8)	383 (9.0)	151 (7.5)	766 (9.1)
Number of glucose-lowering medication (n)				
0	267 (12.4)	584 (13.7)	274 (13.7)	1,125 (13.4)
1	1,395 (64.8)	2,844 (66.7)	1,338 (66.8)	5,577 (66.3)
2	381 (17.7)	659 (15.5)	309 (15.4)	1,349 (16.0)
3+	109 (5.1)	175 (4.1)	82 (4.1)	366 (4.4)

Legend: Continuous variables are presented in median [interquartile range]. Categorical variables presented in numbers (percentage).

ESM table 5: Incident cardiovascular endpoints according to birthweight

Variable		**<3000 g (n=2,152)**	**3000–3700 g (n=4,262)**	**>3700 g (n=2,003)**	**Total (n=8,417)**
Composite CVD					
	No	1,861 (86.5)	3,675 (86.2)	1,694 (84.ø6)	7,230 (85.9)
	Yes	291 (13.5)	587 (13.8)	309 (15.4)	1,187 (14.1)
MACE					
	No	1,932 (89.8)	3,805 (89.3)	1,749 (87.3)	7,486 (88.9)
	Yes	220 (10.2)	457 (10.7)	254 (12.7)	931 (11.1)
Myocardial infarction					
	No	2,049 (95.2)	4,006 (94.0)	1,868 (93.3)	7,923 (94.1)
	Yes	103 (4.8)	256 (6.0)	135 (6.7)	494 (5.9)
Unstable angina pectoris					
	No	2,137 (99.3)	4,220 (99.0)	1,980 (98.9)	8,337 (99.1)
	Yes	15 (0.7)	42 (1.0)	23 (1.2)	80 (1.0)
Stroke					
	No	2,054 (95.5)	4,094 (96.1)	1,923 (96.0)	8,071 (95.9)
	Yes	98 (4.6)	168 (3.9)	80 (4.0)	346 (4.1)
Atrial fibrillation					
	No	2,058 (95.6)	4,013 (94.2)	1,846 (92.2)	7,917 (94.1)
	Yes	94 (4.4)	249 (5.8)	157 (7.8)	500 (5.9)
Heart failure					
	No	2,091 (97.2)	4,115 (96.6)	1,916 (95.7)	8,122 (96.5)
	Yes	61 (2.8)	147 (3.5)	87 (4.3)	295 (3.5)
Peripheral arterial disease					
	No	2,101 (97.6)	4,177 (98.0)	1,964 (98.1)	8,242 (97.9)
	Yes	51 (2.4)	85 (2.0)	39 (1.9)	175 (2.1)
CVD death					
	No	2,091 (97.2)	4,143 (97.2)	1,925 (96.1)	8,159 (96.9)
	Yes	61 (2.8)	119 (2.8)	78 (3.9)	258 (3.1)
All-cause mortality					
	No	1,921 (89.3)	3,709 (87.0)	1,693 (84.5)	7,323 (87.0)
	Yes	231 (10.7)	553 (13.0)	310 (15.5)	1,094 (13.0)

Legend: Variables presented in number (percentages) according to birthweight categories and total.

Abbreviations: CVD = cardiovascular disease, MACE = Major Adverse Cardiovascular Events.

ESM Table 6: Cardiovascular endpoints and comorbidities, prior to DD2 enrolment, according to birthweight

Prior to enrolment	**<3000 g (n=2,152)**	**3000–3700 g (n=4,262)**	**>3700 g (n=2,003)**	**Total (n=8,417)**
Composite CVD				
No	1854 (86.2)	3666 (86.0)	1671 (83.4)	7191 (85.4)
Yes	298 (13.9)	596 (14.0)	332 (16.6)	1226 (14.6)
MACE				
No	1,909 (88.7)	3,770 (88.5)	1,727 (86.2)	7,406 (88.0)
Yes	243 (11.3)	492 (11.5)	276 (13.8)	1,011 (12.0)
Myocardial infarction				
No	1,975 (91.8)	3,913 (91.8)	1,783 (89.0)	7,671 (91.1)
Yes	177 (8.2)	349 (8.2)	220 (11.0)	746 (8.9)
Unstable angina pectoris				
No	2,111 (98.1)	4,180 (98.1)	1,947 (97.2)	8,238 (97.9)
Yes	41 (1.9)	82 (1.9)	56 (2.8)	179 (2.1)
Stroke				
No	2,066 (96.0)	4,107 (96.4)	1,940 (96.9)	8,113 (96.4)
Yes	86 (4.0)	155 (3.6)	63 (3.2)	304 (3.6)
Atrial fibrillation				
No	2,031 (94.4)	3,974 (93.2)	1,833 (91.5)	7,838 (93.1)
Yes	121 (5.6)	288 (6.8)	170 (8.5)	579 (6.9)
Heart failure				
No	2,061 (95.8)	4,085 (95.9)	1,917 (95.7)	8,063 (95.8)
Yes	91 (4.2)	177 (4.2)	86 (4.3)	354 (4.2)
Peripheral arterial disease				
No	2,124 (98.7)	4,203 (98.6)	1,977 (98.7)	8,304 (98.7)
Yes	28 (1.3)	59 (1.4)	26 (1.3)	113 (1.3)
Any cardiovascular disease				
No	1,646 (76.5)	3,141 (73.7)	1,406 (70.2)	6,193 (73.6)
Yes	506 (23.5)	1,121 (26.3)	597 (29.8)	2,224 (26.4)

Legend: Variables presented in number (percentages) according to birthweight categories and total.

Abbreviations: CVD = cardiovascular disease, MACE = Major Adverse Cardiovascular Events.

ESM Table 7: Stepwise adjustments in cox proportional hazard regression of cardiovascular endpoints according to birthweight

Outcome and adjustment	Hazard ratios (95% CI)	
	**<3000 g**	**>3700 g**
**Composite CVD**		
unadjusted	0.99 (0.87, 1.14)	1.13 (0.99, 1.29)
sex + calendar year at birth + family history of type 2 diabetes	1.19 (1.03, 1.36)	0.98 (0.86, 1.12)
+ age at enrolment	1.19 (1.03, 1.36)	0.98 (0.86, 1.12)
+ born-at-term	1.20 (1.03, 1.40)	0.98 (0.86, 1.12)
+ lifestyle (alcohol, smoking, physical activity)	1.21 (1.04, 1.41)	0.96 (0.84, 1.10)
+ marital status + level of urbanization	1.21 (1.04, 1.41)	0.96 (0.84, 1.10)
+ BMI	1.23 (1.05, 1.44)	0.94 (0.82, 1.07)
+ glucose-lowering and antihypertensive medication	1.21 (1.03, 1.41)	0.95 (0.83, 1.09)
**MACE**		
unadjusted	0.97 (0.83, 1.14)	1.20 (1.03, 1.39)
sex + calendar year at birth + family history of type 2 diabetes	1.17 (0.99, 1.37)	1.04 (0.89, 1.21)
+ age at enrolment	1.16 (0.99, 1.37)	1.03 (0.89, 1.21)
+ born-at-term	1.22 (1.01, 1.46)	1.03 (0.89, 1.21)
+ lifestyle (alcohol, smoking, physical activity)	1.23 (1.03, 1.47)	1.02 (0.87, 1.19)
+ marital status + level of urbanization	1.21 (1.02, 1.44)	1.02 (0.88, 1.19)
+ BMI	1.23 (1.03, 1.46)	1.00 (0.86, 1.16)
+ glucose-lowering and antihypertensive medication	1.21 (1.01, 1.43)	1.01 (0.87, 1.17)
**Myocardial infarction**		
unadjusted	0.81 (0.65, 1.02)	1.13 (0.92, 1.39)
sex + calendar year at birth + family history of type 2 diabetes	0.95 (0.76, 1.20)	1.00 (0.81, 1.23)
+ age at enrolment	0.95 (0.75, 1.20)	1.00 (0.81, 1.24)
+ born-at-term	0.97 (0.75, 1.25)	1.00 (0.81, 1.24)
+ lifestyle (alcohol, smoking, physical activity)	0.98 (0.75, 1.27)	0.99 (0.80, 1.23)
+ marital status + level of urbanization	0.97 (0.75, 1.26)	1.00 (0.81, 1.23)
+ BMI	0.98 (0.76, 1.28)	0.98 (0.80, 1.22)
+ glucose-lowering and antihypertensive medication	0.96 (0.74, 1.25)	0.99 (0.80, 1.22)
**Stroke**		
unadjusted	1.19 (0.93, 1.53)	1.02 (0.78, 1.34)
sex + calendar year at birth + family history of type 2 diabetes	1.39 (1.08, 1.78)	0.91 (0.69, 1.19)
+ age at enrolment	1.39 (1.08, 1.79)	0.90 (0.69, 1.18)
+ born-at-term	1.44 (1.09, 1.90)	0.90 (0.69, 1.18)
+ lifestyle (alcohol, smoking, physical activity)	1.45 (1.10, 1.92)	0.88 (0.68, 1.16)
+ marital status + level of urbanization	1.46 (1.11, 1.93)	0.96 (0.74, 1.24)
+ BMI	1.46 (1.11, 1.93)	0.95 (0.73, 1.24)
+ glucose-lowering and antihypertensive medication	1.46 (1.10, 1.92)	0.97 (0.74, 1.26)
**Atrial fibrillation**		
unadjusted	0.76 (0.60, 0.97)	1.38 (1.13, 1.68)
sex + calendar year at birth + family history of type 2 diabetes	0.90 (0.70, 1.14)	1.19 (0.97, 1.46)
+ age at enrolment	0.90 (0.71, 1.14)	1.20 (0.98, 1.47)
+ born-at-term	0.82 (0.63, 1.09)	1.21 (0.99, 1.48)

+ lifestyle (alcohol, smoking, physical activity)	0.82 (0.62, 1.08)	1.20 (0.98, 1.47)
+ marital status + level of urbanization	0.77 (0.59, 1.00)	1.19 (0.98, 1.45)
+ BMI	0.81 (0.62, 1.06)	1.12 (0.92, 1.37)
+ glucose-lowering and antihypertensive medication	0.81 (0.61, 1.06)	1.13 (0.93, 1.38)
**Heart failure**		
unadjusted	0.84 (0.62, 1.13)	1.28 (0.98, 1.67)
sex + calendar year at birth + family history of type 2 diabetes	1.00 (0.74, 1.35)	1.09 (0.83, 1.43)
+ age at enrolment	1.00 (0.74, 1.36)	1.08 (0.83, 1.42)
+ born-at-term	0.97 (0.69, 1.36)	1.08 (0.83, 1.42)
+ lifestyle (alcohol, smoking, physical activity)	0.95 (0.68, 1.34)	1.05 (0.80, 1.38)
+ marital status + level of urbanization	0.89 (0.64, 1.25)	1.03 (0.79, 1.34)
+ BMI	0.95 (0.68, 1.34)	0.94 (0.72, 1.23)
+ glucose-lowering and antihypertensive medication	0.93 (0.66, 1.31)	0.95 (0.72, 1.24)
**Peripheral arterial disease**		
Unadjusted	1.23 (0.86, 1.74)	0.98 (0.67, 1.44)
sex + calendar year at birth + family history of type 2 diabetes	1.49 (1.05, 2.13)	0.83 (0.56, 1.22)
+ age at enrolment	1.48 (1.04, 2.11)	0.84 (0.57, 1.23)
+ born-at-term	1.34 (0.89, 2.01)	0.84 (0.57, 1.23)
+ lifestyle (alcohol, smoking, physical activity)	1.37 (0.91, 2.05)	0.82 (0.56, 1.21)
+ marital status + level of urbanization	1.37 (0.92, 2.06)	0.81 (0.55, 1.19)
+ BMI	1.34 (0.89, 2.02)	0.84 (0.57, 1.24)
+ glucose-lowering and antihypertensive medication	1.28 (0.85, 1.93)	0.85 (0.57, 1.25)
**CVD death**		
unadjusted	1.01 (0.79, 1.30)	1.31 (1.04, 1.66)
sex + calendar year at birth + family history of type 2 diabetes	1.26 (0.99, 1.62)	1.09 (0.86, 1.37)
+ age at enrolment	1.28 (0.99, 1.64)	1.09 (0.86, 1.38)
+ born-at-term	1.36 (1.03, 1.79)	1.09 (0.86, 1.38)
+ lifestyle (alcohol, smoking, physical activity)	1.39 (1.05, 1.82)	1.06 (0.84, 1.35)
+ marital status + level of urbanization	1.39 (1.06, 1.84)	1.06 (0.83, 1.34)
+ BMI	1.44 (1.09, 1.90)	1.00 (0.79, 1.27)
+ glucose-lowering and antihypertensive medication	1.40 (1.06, 1.85)	1.01 (0.80, 1.28)
**CVD death – without stroke**		
unadjusted	1.04 (0.81, 1.34)	1.29 (1.01, 1.65)
sex + calendar year at birth + family history of type 2 diabetes	1.31 (1.01, 1.70)	1.06 (0.83, 1.35)
+ age at enrolment	1.32 (1.02, 1.71)	1.06 (0.83, 1.35)
+ born-at-term	1.41 (1.06, 1.87)	1.06 (0.83, 1.35)
+ lifestyle (alcohol, smoking, physical activity)	1.43 (1.08, 1.90)	1.04 (0.81, 1.32)
+ marital status + level of urbanization	1.44 (1.08, 1.92)	1.03 (0.80, 1.32)
+ BMI	1.50 (1.12, 1.99)	0.97 (0.76, 1.25)
+ glucose-lowering and antihypertensive medication	1.44 (1.08, 1.92)	0.98 (0.76, 1.25)
**All-cause mortality**		
unadjusted	0.85 (0.73, 0.99)	1.20 (1.05, 1.38)
sex + calendar year at birth + family history of type 2 diabetes	1.04 (0.89, 1.22)	0.99 (0.86, 1.15)
+ age at enrolment	1.04 (0.89, 1.22)	0.99 (0.86, 1.14)

+ born-at-term	1.06 (0.89, 1.26)	0.99 (0.86, 1.14)
+ lifestyle (alcohol, smoking, physical activity)	1.07 (0.90, 1.27)	0.97 (0.84, 1.12)
+ marital status + level of urbanization	1.07 (0.91, 1.27)	0.96 (0.84, 1.10)
+ BMI	1.07 (0.91, 1.27)	0.96 (0.84, 1.10)
+ glucose-lowering and antihypertensive medication	1.05 (0.89, 1.25)	0.96 (0.84, 1.10)

Legend: hazard ratios comparing reference birthweight of 3000-3700 g vs. the lower birthweight category (<3000 g) and the higher birthweight category (>3700 g). Abbreviations: CI = confidence interval, CVD = cardiovascular disease, MACE = major adverse cardiovascular events, BMI = body mass index

ESM Table 8: Stepwise adjustments in cox proportional hazard regression models of cardiovascular endpoints according to conventional clinically defined birthweights.

Outcome and adjustment	Hazard ratios (95% CI)	
	**<2500 g**	**>4500 g**
**Composite CVD**		
unadjusted	1.00 (0.80, 1.26)	1.00 (0.63, 1.57)
sex + calendar year at birth + family history of type 2 diabetes + age at enrolment	1.28 (1.02, 1.61)	0.92 (0.58, 1.45)
+ born-at-term	1.29 (0.98, 1.70)	0.92 (0.58, 1.45)
**MACE**		
unadjusted	0.87 (0.65, 1.15)	0.94 (0.56, 1.60)
sex + calendar year at birth + family history of type 2 diabetes + age at enrolment	1.11 (0.84, 1.47)	0.88 (0.52, 1.50)
+ born-at-term	1.20 (0.85, 1.68)	0.89 (0.52, 1.50)
**Myocardial infarction**		
unadjusted	1.05 (0.73, 1.49)	0.94 (0.45, 2.00)
sex + calendar year at birth + family history of type 2 diabetes + age at enrolment	1.26 (0.88, 1.80)	0.87 (0.41, 1.85)
+ born-at-term	1.52 (0.98, 2.36)	0.87 (0.41, 1.84)
**Stroke**		
unadjusted	0.72 (0.44, 1.20)	0.90 (0.37, 2.20)
sex + calendar year at birth + family history of type 2 diabetes + age at enrolment	0.91 (0.55, 1.52)	0.89 (0.37, 2.16)
+ born-at-term	0.74 (0.41, 1.33)	0.88 (0.36, 2.15)
**Atrial fibrillation**		
unadjusted	0.60 (0.39, 0.95)	0.78 (0.35, 1.74)
sex + calendar year at birth + family history of type 2 diabetes + age at enrolment	0.77 (0.49, 1.21)	0.70 (0.31, 1.57)
+ born-at-term	0.65 (0.39, 1.09)	0.70 (0.31, 1.57)
**Heart failure**		
unadjusted	0.79 (0.47, 1.33)	0.67 (0.21, 2.11)
sex + calendar year at birth + family history of type 2 diabetes + age at enrolment	1.03 (0.61, 1.75)	0.59 (0.19, 1.84)
+ born-at-term	0.98 (0.52, 1.82)	0.59 (0.19, 1.84)
**Peripheral arterial disease**		
unadjusted	1.83 (1.13, 2.96)	1.63 (0.60, 4.43)
sex + calendar year at birth + family history of type 2 diabetes + age at enrolment	2.39 (1.47, 3.88)	1.47 (0.54, 4.00)
+ born-at-term	2.17 (1.15, 4.07)	1.47 (0.54, 3.99)
**CVD death**		

unadjusted	0.67 (0.38, 1.20)	1.44 (0.64, 3.24)
sex + calendar year at birth + family history of type 2 diabetes + age at enrolment	0.94 (0.52, 1.68)	1.30 (0.57, 2.93)
+ born-at-term	1.15 (0.57, 2.31)	1.30 (0.57, 2.94)
**All-cause mortality**		
unadjusted	0.80 (0.61, 1.05)	0.99 (0.61, 1.63)
sex + calendar year at birth + family history of type 2 diabetes + age at enrolment	1.08 (0.82, 1.42)	0.90 (0.55, 1.47)
+ born-at-term	1.15 (0.83, 1.60)	0.90 (0.55, 1.47)

Legend: hazard ratios comparing reference birthweight of 2500-4500 g vs. the lower birthweight category (<2500 g) and the higher birthweight category (>4500 g). Abbreviations: CI = confidence interval, CVD = cardiovascular disease, MACE = major adverse cardiovascular events.

ESM Table 9: Stepwise adjustments in cox proportional hazard regression models of cardiovascular endpoints according to birthweight without any pre-existing cardiovascular disease.

Outcome and adjustment	Hazard ratios (95% CI)	
	**<3000 g**	**>3700 g**
**Composite CVD**		
unadjusted	0.98 (0.80, 1.19)	1.16 (0.95, 1.41)
sex + calendar year at birth + family history of type 2 diabetes + age at enrolment	1.14 (0.93, 1.39)	1.03 (0.85, 1.25)
+ born-at-term	1.09 (0.87, 1.36)	1.03 (0.85, 1.25)
**MACE**		
unadjusted	0.97 (0.77, 1.22)	1.20 (0.96, 1.51)
sex + calendar year at birth + family history of type 2 diabetes + age at enrolment	1.12 (0.89, 1.42)	1.07 (0.85, 1.34)
+ born-at-term	1.11 (0.85, 1.45)	1.07 (0.85, 1.34)
**Myocardial infarction**		
unadjusted	0.67 (0.48, 0.95)	1.14 (0.84, 1.54)
sex + calendar year at birth + family history of type 2 diabetes + age at enrolment	0.78 (0.55, 1.10)	1.03 (0.76, 1.39)
+ born-at-term	0.79 (0.53, 1.16)	1.02 (0.76, 1.39)
**Stroke**		
unadjusted	1.41 (1.00, 1.98)	1.22 (0.84, 1.77)
sex + calendar year at birth + family history of type 2 diabetes + age at enrolment	1.61 (1.14, 2.28)	1.08 (0.74, 1.58)
+ born-at-term	1.50 (1.01, 2.22)	1.09 (0.74, 1.58)
**Atrial fibrillation**		
unadjusted	0.78 (0.58, 1.05)	1.39 (1.08, 1.79)
sex + calendar year at birth + family history of type 2 diabetes + age at enrolment	0.91 (0.68, 1.23)	1.23 (0.95, 1.59)
+ born-at-term	0.76 (0.54, 1.08)	1.24 (0.96, 1.60)
**Heart failure**		
unadjusted	0.89 (0.59, 1.34)	1.01 (0.67, 1.52)
sex + calendar year at birth + family history of type 2 diabetes + age at enrolment	1.03 (0.68, 1.56)	0.89 (0.59, 1.34)
+ born-at-term	1.05 (0.66, 1.66)	0.89 (0.59, 1.34)
**Peripheral arterial disease**		
unadjusted	1.03 (0.55, 1.95)	0.94 (0.47, 1.88)
sex + calendar year at birth + family history of type 2 diabetes + age at enrolment	1.26 (0.66, 2.40)	0.80 (0.40, 1.61)
+ born-at-term	1.12 (0.53, 2.38)	0.81 (0.40, 1.62)
**CVD death**		

unadjusted	0.88 (0.57, 1.35)	1.37 (0.93, 2.02)
sex + calendar year at birth + family history of type 2 diabetes + age at enrolment	1.02 (0.66, 1.56)	1.21 (0.82, 1.78)
+ born-at-term	0.99 (0.61, 1.60)	1.21 (0.82, 1.79)
**All-cause mortality**		
unadjusted	0.91 (0.74, 1.12)	1.14 (0.93, 1.39)
sex + calendar year at birth + family history of type 2 diabetes + age at enrolment	1.08 (0.88. 1.33)	0.97 (0.80, 1.19)
+ born-at-term	1.06 (0.84,1.34)	0.97 (0.80,1.19)

Legend: hazard ratios comparing reference birthweight of 3000-3700 g vs. the lower birthweight category (<3000 g) and the higher birthweight category (>3700 g). Abbreviations: CI = confidence interval, CVD = cardiovascular disease, MACE = major adverse cardiovascular events.

ESM Table 10: Stepwise adjustments in Fine and Gray sub-distributional models of cardiovascular endpoints according to birthweight

Outcome and adjustment	Sub-distributional hazard ratios (95% CI)	
	**<3000 g**	**>3700 g**
**Composite CVD**		
unadjusted	1.01 (0.88, 1.15)	1.12 (0.98, 1.28)
sex + calendar year at birth + family history of type 2 diabetes	1.20 (1.04, 1.37)	0.99 (0.86, 1.13)
+ age at enrolment	1.20 (1.04, 1.37)	0.98 (0.86, 1.13)
+ born-at-term	1.21 (1.04, 1.41)	0.98 (0.86, 1.13)
**MACE**		
unadjusted	0.99 (0.85, 1.15)	1.19 (1.02, 1.37)
sex + calendar year at birth + family history of type 2 diabetes	1.18 (1.01, 1.38)	1.04 (0.9, 1.21)
+ age at enrolment	1.18 (1.01, 1.38)	1.04 (0.9, 1.21)
+ born-at-term	1.21 (1.02, 1.44)	1.04 (0.9, 1.21)
**Myocardial infarction**		
unadjusted	0.82 (0.65, 1.03)	1.12 (0.91, 1.38)
sex + calendar year at birth + family history of type 2 diabetes	0.96 (0.76, 1.21)	1.00 (0.81, 1.23)
+ age at enrolment	0.95 (0.76, 1.20)	1.00 (0.81, 1.23)
+ born-at-term	0.98 (0.75, 1.27)	1.00 (0.81, 1.23)
**Stroke**		
unadjusted	1.22 (0.96, 1.56)	1.08 (0.84, 1.40)
sex + calendar year at birth + family history of type 2 diabetes	1.41 (1.10, 1.81)	0.97 (0.75, 1.26)
+ age at enrolment	1.41 (1.10, 1.82)	0.97 (0.75, 1.26)
+ born-at-term	1.45 (1.10, 1.91)	0.97 (0.75, 1.26)
**Atrial fibrillation**		
unadjusted	0.75 (0.59, 0.94)	1.33 (1.09, 1.61)
sex + calendar year at birth + family history of type 2 diabetes	0.86 (0.68, 1.09)	1.17 (0.96, 1.43)
+ age at enrolment	0.86 (0.68, 1.09)	1.17 (0.96, 1.43)
+ born-at-term	0.76 (0.58, 1.00)	1.18 (0.97, 1.43)
**Heart failure**		
unadjusted	0.86 (0.64, 1.14)	1.23 (0.95, 1.60)
sex + calendar year at birth + family history of type 2 diabetes	1.00 (0.74, 1.33)	1.08 (0.83, 1.41)
+ age at enrolment	1.00 (0.75, 1.34)	1.08 (0.83, 1.40)
+ born-at-term	0.90 (0.64, 1.28)	1.08 (0.83, 1.41)
**Peripheral arterial disease**		
Unadjusted	1.24 (0.88, 1.76)	0.97 (0.67, 1.42)
sex + calendar year at birth + family history of type 2 diabetes	1.50 (1.06, 2.12)	0.83 (0.57, 1.22)
+ age at enrolment	1.49 (1.05, 2.11)	0.83 (0.57, 1.22)
+ born-at-term	1.35 (0.90, 2.00)	0.84 (0.57, 1.22)
**CVD death**		
unadjusted	1.03 (0.81, 1.32)	1.30 (1.03, 1.64)
sex + calendar year at birth + family history of type 2 diabetes	1.28 (1.00, 1.64)	1.09 (0.86, 1.38)
+ age at enrolment	1.29 (1.01, 1.66)	1.09 (0.86, 1.38)
+ born-at-term	1.39 (1.06, 1.83)	1.09 (0.86, 1.38)

Legend: sub-distributional hazard ratios comparing reference birthweight of 3000-3700 g vs. the lower birthweight category (<3000 g) and the higher birthweight category (>3700 g). Abbreviations: CI = confidence interval, CVD = cardiovascular disease, MACE = major adverse cardiovascular events

ESM Table 11: Cardiovascular endpoints according to birthweight: Results stratified by sex, restricted to individuals born at term, and stratified by calendar year of birth, respectively.

Hazard ratios (95% CI)

**Male**		**Female**		**Born at term**		**Year at birth 1920-1953**	**Year at birth 1954+**
**Composite CVD**							
**<3100 g**	1.25 (1.06, 1.48)	**<3000 g**	1.10 (0.84, 1.44)	**<3000 g**	1.18 (1.01, 1.39)	1.21 (1.01, 1.46)	1.17 (0.89, 1.53)
**>3750 g**	0.96 (0.81, 1.13)	**>3600 g**	1.04 (0.80, 1.34)	**>3700 g**	0.97 (0.85, 1.11)	1.00 (0.86, 1.16)	0.91 (0.69, 1.22)
**MACE**							
**<3100 g**	1.31 (1.09, 1.58)	**<3000 g**	1.00 (0.73, 1.37)	**<3000 g**	1.17 (0.98, 1.40)	1.22 (0.99, 1.50)	1.14 (0.84, 1.53)
**>3750 g**	1.00 (0.83, 1.19)	**>3600 g**	1.09 (0.82, 1.45)	**>3700 g**	1.03 (0.89, 1.20)	1.08 (0.91, 1.27)	0.96 (0.70, 1.30)
**Myocardial infarction**							
**<3100 g**	1.21 (0.93, 1.57)	**<3000 g**	0.81 (0.49, 1.36)	**<3000 g**	0.97 (0.74, 1.27)	0.88 (0.62, 1.25)	1.10 (0.74, 1.64)
**>3750 g**	1.07 (0.84, 1.37)	**>3600 g**	0.73 (0.44, 1.21)	**>3700 g**	1.00 (0.80, 1.23)	0.99 (0.77, 1.27)	1.05 (0.71, 1.53)
**Stroke**							
**<3100 g**	1.55 (1.13, 2.12)	**<3000 g**	1.00 (0.62, 1.62)	**<3000 g**	1.40 (1.05, 1.86)	1.42 (1.01, 1.97)	1.49 (0.91, 2.45)
**>3750 g**	0.84 (0.60, 1.17)	**>3600 g**	1.07 (0.69, 1.66)	**>3700 g**	0.97 (0.75, 1.26)	1.06 (0.79, 1.42)	0.71 (0.38, 1.33)
**Atrial fibrillation**							
**<3100 g**	0.86 (0.63, 1.16)	**<3000 g**	0.79 (0.52, 1.20)	**<3000 g**	0.78 (0.59, 1.04)	0.73 (0.54, 1.00)	0.93 (0.53, 1.65)
**>3750 g**	1.04 (0.81, 1.33)	**>3600 g**	1.48 (1.07, 2.04)	**>3700 g**	1.18 (0.97, 1.44)	1.18 (0.96, 1.46)	1.35 (0.83, 2.21)
**Heart failure**							
**<3100 g**	0.95 (0.65, 1.39)	**<3000 g**	1.13 (0.66, 1.94)	**<3000 g**	0.97 (0.69, 1.38)	1.00 (0.68, 1.48)	0.74 (0.38, 1.43)
**>3750 g**	1.10 (0.80, 1.52)	**>3600 g**	1.18 (0.72, 1.94)	**>3700 g**	1.09 (0.84, 1.43)	0.99 (0.73, 1.34)	1.45 (0.84, 2.50)
**Peripheral arterial disease**							
**<3100 g**	1.12 (0.69, 1.82)	**>3600 g**	1.60 (0.82, 3.10)	**<3000 g**	1.32 (0.87, 2.02)	1.39 (0.88, 2.20)	1.14 (0.46, 2.83)
**>3750 g**	1.00 (0.64, 1.55)	**>3600 g**	0.53 (0.22, 1.28)	**>3700 g**	0.81 (0.55, 1.20)	0.84 (0.55, 1.28)	0.91 (0.34, 2.41)

			**CVD death**				
**<3100 g**	1.36 (1.00, 1.84)	**<3000 g**	1.37 (0.84, 2.23)	**<3000 g**	1.32 (1.00, 1.75)	1.47 (1.09, 2.00)	1.07 (0.57, 1.99)
**>3750 g**	0.90 (0.67, 1.20)	**>3600 g**	1.61 (1.05, 2.48)	**>3700 g**	1.08 (0.85, 1.36)	1.09 (0.84, 1.41)	1.23 (0.68, 2.20)
			**All-cause mortality**				
**<3100 g**	0.99 (0.82, 1.20)	**<3000 g**	1.07 (0.83, 1.39)	**<3000 g**	1.02 (0.86, 1.21)	1.03 (0.86, 1.24)	1.27 (0.88, 1.83)
**>3750 g**	0.99 (0.84, 1.16)	**>3600 g**	1.02 (0.80, 1.30)	**>3700 g**	0.99 (0.86, 1.13)	0.95 (0.82, 1.10)	1.45 (1.03, 2.04)

Legend: hazard ratios of stratified analysis. Sex stratified analysis adjusted for calendar year at birth, age at enrolment, family history of type 2 diabetes, and born-at-term status. Born-at-term stratified analysis adjusted for sex, calendar year at birth, age at enrolment, and family history of type 2 diabetes. Year at birth stratified analysis adjusted for sex, age at enrolment, family history of type 2 diabetes, and born-at-term status.
Abbreviations: CI = confidence interval, CVD = cardiovascular disease, MACE = major adverse cardiovascular events, BMI = body mass index

<u>ESM Figures</u>

ESM Fig. 1: Directed acyclic graph

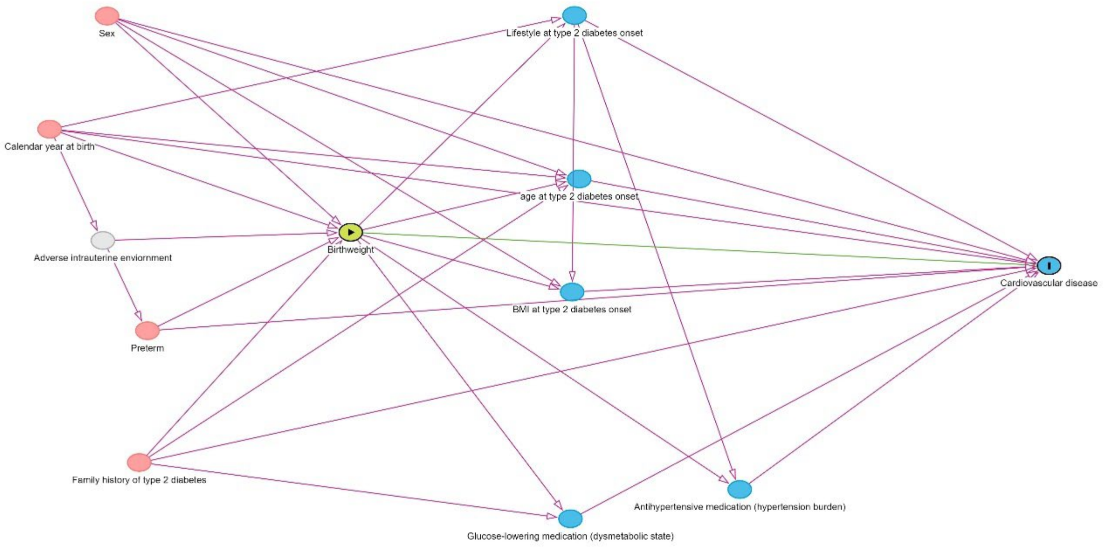

Legend: Directed acyclic graph depicting the potential causal relationship between birthweight and cardiovascular disease in people with type 2 diabetes.
Exposure: birthweight
Outcome: cardiovascular disease
Confounders: preterm birth, family history of type 2 diabetes, sex, calendar year at birth.

Total effect: minimal sufficient adjustment sets for estimating the total effect of birthweight on cardiovascular disease: Sex, calendar year at birth, family history of 2 diabetes, born-at-term status (preterm)

Direct effect: minimal sufficient adjustment sets for estimating the direct effect of birthweight on cardiovascular disease: sex, calendar year at birth, family history of 2 diabetes, born-at-term status (preterm), age at type 2 diabetes onset, lifestyle (physical activity, alcohol consumption, smoking status), BMI, antihypertensive medication, and glucose-lowering medication.

ESM Fig. 2: Flowchart of the study population

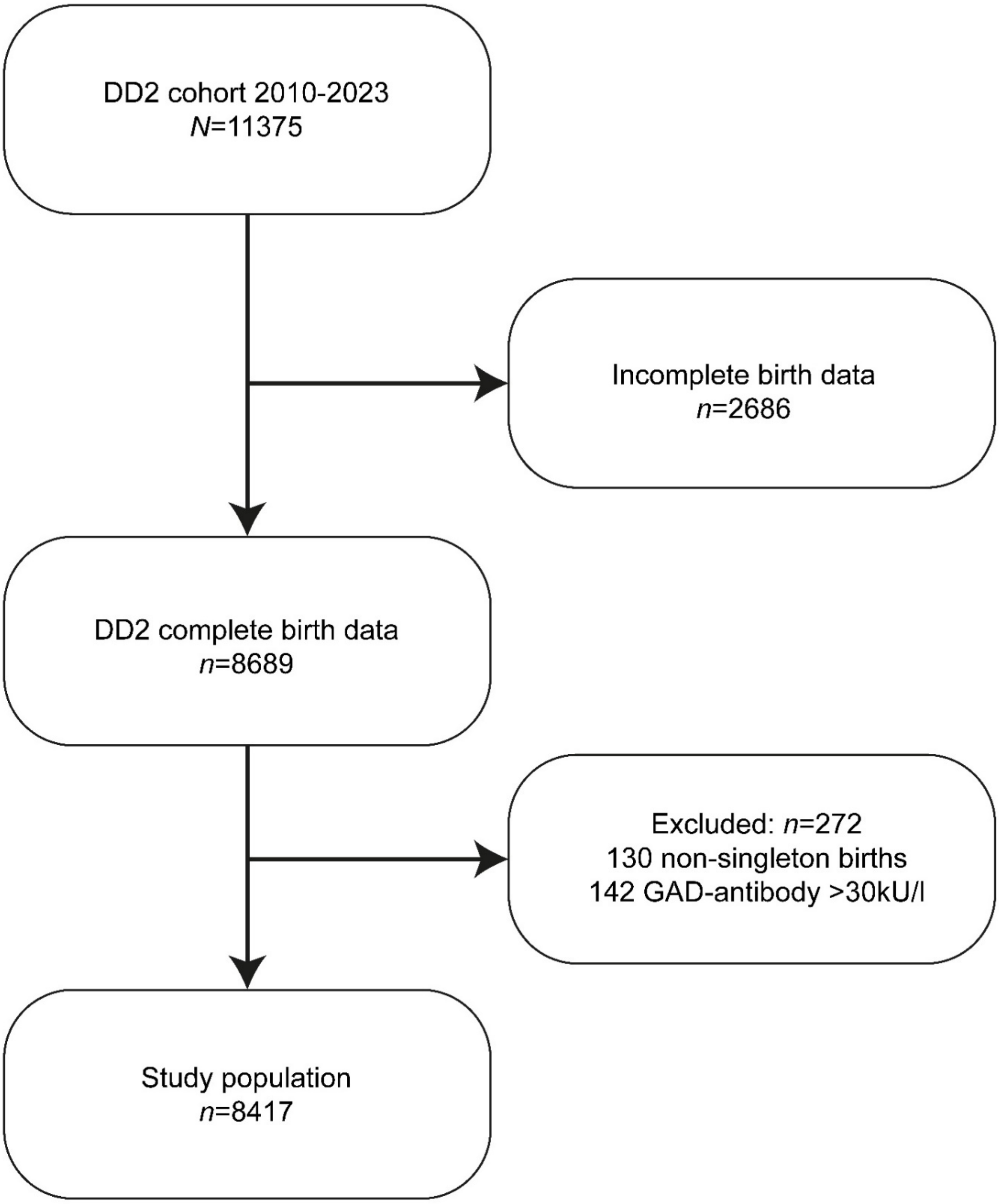

ESM Fig. 3: Ten-year standardized risk and risk differences for cardiovascular endpoints according to birthweight, without pre-existing cardiovascular disease

Outcome	Events/at risk	10-year risk, % (95% CI)	RD, % (95% CI)
Composite CVD			
<3000 g	148/1646	12.0 (10.0, 14.1)	0.8 (−1.6,3.2)
3000–3700 g	300/3141	11.1 (9.9, 12.5)	Ref
>3700 g	153/1406	11.5 (9.8, 13.3)	0.4 (−1.6,2.4)
MACE			
<3000 g	98/1646	9.0 (7.2, 10.8)	0.8 (−1.3,3.1)
3000–3700 g	211/3141	8.1 (7.0, 9.3)	Ref
>3700 g	114/1406	8.7 (7.2, 10.4)	0.6 (−1.2,2.4)
MI			
<3000 g	44/1646	3.8 (2.6, 5.0)	−1.0 (−2.5,0.5)
3000–3700 g	129/3141	4.8 (3.9, 5.7)	Ref
>3700 g	65/1406	4.9 (3.7, 6.2)	0.1 (−1.3,1.6)
Stroke			
<3000 g	49/1646	4.6 (3.2, 6.0)	1.5 (−0.1,3.1)
3000–3700 g	76/3141	3.1 (2.4, 3.9)	Ref
>3700 g	42/1406	3.4 (2.4, 4.5)	0.3 (−0.9,1.5)
Afib			
<3000 g	57/1646	4.5 (3.4, 5.7)	−1.4 (−2.9,0.2)
3000–3700 g	153/3141	5.8 (4.9, 6.8)	Ref
>3700 g	94/1406	7.2 (5.7, 8.7)	1.3 (−0.3,3.0)
HF			
<3000 g	33/1646	3.0 (1.9, 4.3)	0.1 (−1.3,1.6)
3000–3700 g	73/3141	2.9 (2.3, 3.7)	Ref
>3700 g	33/1406	2.6 (1.8, 3.6)	−0.3 (−1.4,0.8)
PAD			
<3000 g	15/1646	1.1 (0.5, 1.9)	0.1 (−0.7,1.0)
3000–3700 g	24/3141	1.0 (0.6, 1.5)	Ref
>3700 g	9/1406	0.8 (0.4, 1.4)	−0.2 (−0.8,0.4)
CVD death			
<3000 g	18/1646	1.6 (0.9, 2.4)	0.2 (−0.6,1.2)
3000–3700 g	35/3141	1.3 (0.9, 1.8)	Ref
>3700 g	23/1406	1.5 (0.9, 2.2)	0.2 (−0.5,1.0)
All-cause mortality			
<3000 g	119/1646	11.2 (9.3, 13.1)	0.6 (−1.6,2.8)
3000–3700 g	270/3141	10.6 (9.4, 11.8)	Ref
>3700 g	138/1406	10.3 (8.7, 12.0)	−0.2 (−2.1,1.7)

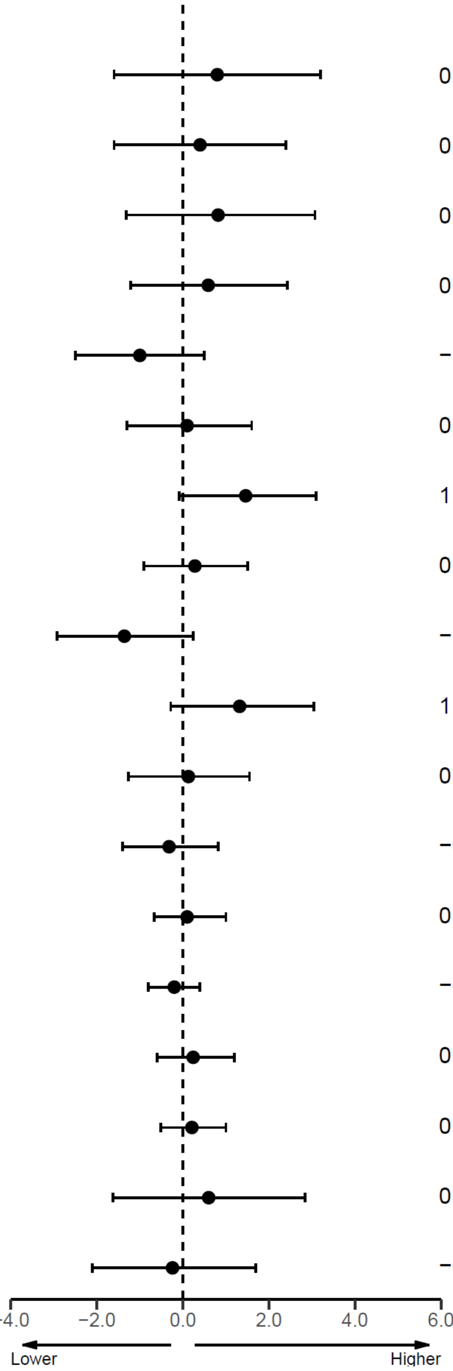

Legend: Forest plot depicting 10-year standardized risks and risk differences for different endpoints according to birthweight groups without pre-existing CVD. Standardized to the distribution of sex, age at DD2 enrolment, calendar year of birth, family history of type 2 diabetes, and born-at-term status. Composite CVD endpoint includes MI, stroke, unstable angina pectoris, coronary or peripheral revascularization, HF hospitalization, or CVD death. MACE includes MI and coronary revascularization, stroke, or CVD death. Abbreviations: CI = confidence interval; CVD = cardiovascular disease; MACE = major adverse cardiovascular event; MI = myocardial infarction; Afib = atrial fibrillation; HF = heart failure; PAD = peripheral arterial disease; RD = risk difference.

ESM references

[1] Christensen DH, Nicolaisen SK, Berencsi K, et al. (2018) Danish Centre for Strategic Research in Type 2 Diabetes (DD2) project cohort of newly diagnosed patients with type 2 diabetes: a cohort profile. BMJ Open 8(4): e017273. 10.1136/bmjopen-2017-017273
[2] van Buuren S, Groothuis-Oudshoorn K (2011) mice: Multivariate Imputation by Chained Equations in R. Journal of Statistical Software 45(3): 1 - 67. 10.18637/jss.v045.i03
[3] Hansen AL, Thomsen RW, Brøns C, et al. (2023) Birthweight is associated with clinical characteristics in people with recently diagnosed type 2 diabetes. Diabetologia. 10.1007/s00125-023-05936-1
[4] Christensen H, Nielsen JS, Sørensen KM, Melbye M, Brandslund I (2012) New national Biobank of The Danish Center for Strategic Research on Type 2 Diabetes (DD2). Clin Epidemiol 4: 37-42. 10.2147/clep.S33042
[5] Nielsen JS, Thomsen RW, Steffensen C, Christiansen JS (2012) The Danish Centre for Strategic Research in Type 2 Diabetes (DD2) study: implementation of a nationwide patient enrollment system. Clin Epidemiol 4(Suppl 1): 27-36. 10.2147/clep.S30838
[6] Jørgensen ME, Kristensen JK, Reventlov Husted G, Cerqueira C, Rossing P (2016) The Danish Adult Diabetes Registry. Clin Epidemiol 8: 429-434. 10.2147/clep.S99518
[7] Lynge E, Sandegaard JL, Rebolj M (2011) The Danish National Patient Register. Scand J Public Health 39(7 Suppl): 30-33. 10.1177/1403494811401482
[8] Pottegård A, Schmidt SAJ, Wallach-Kildemoes H, Sørensen HT, Hallas J, Schmidt M (2017) Data Resource Profile: The Danish National Prescription Registry. Int J Epidemiol 46(3): 798-798f. 10.1093/ije/dyw213
[9] Schmidt M, Pedersen L, Sørensen HT (2014) The Danish Civil Registration System as a tool in epidemiology. Eur J Epidemiol 29(8): 541-549. 10.1007/s10654-014-9930-3
[10] Helweg-Larsen K (2011) The Danish Register of Causes of Death. Scand J Public Health 39(7 Suppl): 26-29. 10.1177/1403494811399958
[11] Bliddal M, Broe A, Pottegård A, Olsen J, Langhoff-Roos J (2018) The Danish Medical Birth Register. Eur J Epidemiol 33(1): 27-36. 10.1007/s10654-018-0356-1
[12] Hill NR, Levy JC, Matthews DR (2013) Expansion of the homeostasis model assessment of β-cell function and insulin resistance to enable clinical trial outcome modeling through the interactive adjustment of physiology and treatment effects: iHOMA2. Diabetes Care 36(8): 2324-2330. 10.2337/dc12-0607
[13] Stone CJ (1986) [Generalized Additive Models]: Comment. Statistical Science 1(3): 312-314
[14] Harrell FE (2016) Regression modeling strategies. Springer International Publishing, Cham, Switzerland
